# An assessment of contaminants and benthic condition in the Matagorda Bay system (Texas, USA)

**DOI:** 10.1007/s11356-026-37918-2

**Published:** 2026-06-15

**Authors:** Jasmine J. Caillier, Paul A. Montagna, Marie E. DeLorenzo, Katy W. Chung, Peter B. Key

**Affiliations:** 1 Department of Biological Sciences, University of South Carolina, Columbia, SC USA; 2 Texas A&M University-Corpus Christi, Harte Research Institute, Corpus Christi, TX USA; 3 National Oceanic and Atmospheric Administration, National Ocean Service, Charleston, SC USA

**Keywords:** Benthic macrofauna, Trace elements, Sediment chemistry, Persistent organic pollutants, Lavaca Bay, Matagorda Bay, Sediment quality triad

## Abstract

The Matagorda Bay system has suffered a long-term decline in benthic abundance, biomass, and diversity since the 1980’s. This study examined sediment contamination to determine if legacy pollutants are degrading sediment quality and are a possible cause for ecosystem degradation. Degradation can be indicated by a decline in benthic integrity (i.e., diversity), decreased survival rates of organisms exposed to sediment, and sediment chemical contaminant concentrations over threshold limits. These methods form the Sediment Quality Triad to assess environmental effects. There were no contaminant concentrations above threshold limits for PCBs or DDTs, but 46% of 24 stations had concentrations over threshold limits, established by sediment quality guidelines, for seven trace elements and one PAH: arsenic (As), cadmium (Cd), mercury (Hg), copper (Cu), lead (Pb), nickel (Ni), and silver (Ag); and dibenzo (a,h) anthracene. Mostly near river mouths, 16 of the 24 stations had moderate to high toxicity, and 17 out of the 24 stations had fair to low diversity. Toxicity was inversely correlated with diversity, but there were no correlations between sediment chemistry and toxicity or benthic metrics. These findings indicate that legacy chemical contamination is not driving estuary-wide degradation of benthic communities The only station where high contamination was linked to high toxicity and low diversity was in the uppermost region of Lavaca Bay close to creek mouths. Low diversity and/or high toxicity was common near river and creek mouths. Because unmeasured pollution could be entering from rivers and creeks, management plans for the watershed and non-point sources could help to protect this ecosystem.

**Supplementary Information:**

The online version contains supplementary material available at 10.1007/s11356-026-37918-2.

## Introduction

The Matagorda Bay system (MBS), which is also known as Lavaca-Colorado Estuary, is one of seven major estuarine systems on the Texas coast and provides agricultural, industrial, residential, and recreational benefits (Ward and Armstrong 1980; Montagna et al. 2013; 2025). It is the second largest estuary in Texas and is comprised of Lavaca Bay and Matagorda Bay, in addition to four smaller bays: Keller, Carancahua, Chocolate, and Tres Palacios Bays (TDWR 1980). Lavaca Bay is the main source of freshwater inflow to Matagorda Bay because it is connected to the Lavaca River. Freshwater inflow is vital to the health of the ecosystems and species living in the estuary (Pollack et al. 2011). Lavaca and Matagorda Bays provide critical feeding, habitat, and nursery areas for various pelagic and benthic species. Specifically, benthic organisms are important to maintain sediment and water quality, and function as a food source for many other species in the bay.

For the last 30 years, the Matagorda Bay system has been suffering from ecosystem degradation as indicated by declines in benthic macroinvertebrate abundance, biomass, and diversity (Pollack et al. 2011; Montagna et al. 2025). Benthic organisms are a key bioindicator of degradation because they tend to be sessile, very abundant, highly diverse, and long-lived relative to plankton (Dauer 1993). Estuaries receive freshwater inputs from bayous, creeks, and rivers, which can carry pollutants via runoff from watersheds or permitted discharges (Kennish 2002; Montagna et al. 2013). Trace elements and persistent chemical contaminants from point and nonpoint sources could be deposited in estuarine sediments where benthic organisms are directly exposed (Baker 1992). Such a pollution pathway could be causing this observed decline in the condition of the estuary.

In the MBS, there are two known industrial point sources directly discharging various contaminants into the bay: Formosa Plastics Co. (Carr et al. 2001) and the former aluminum smelting ALCOA plant, a superfund site that shut down smelting operations in 1980, ceased alumina refining in June 2016, and permanently closed in 2019 (ALCOA 2025; Bisset et al. 2008; U.S. EPA 2023). In 2001, the EPA signed a record of decision to address mercury and PAH contaminated soil from the industrial plant (U.S. EPA 2023). Studies have been conducted in this estuary, but they were mainly focused on either natural stressors (e.g., temperature, salinity and nutrient fluctuations, changes in dissolved oxygen, freshwater inflow etc.) or effects of the ALCOA discharges (Carr et al. 2001; Pollack et al. 2011). Thus, a holistic approach is needed to determine if pollution-induced degradation of sediment quality and the benthic community is occurring systemwide in the MBS.

The purpose of the current study is to assess the chemical and biological condition of the MBS, focusing on the effects of legacy contaminants on benthic organisms. This study will answer environmental questions about the MBS: Are legacy pollutants contributing to the degradation of sediment quality? If so, which locations are most affected, and what common characteristics do these sites share? Is there greater contamination in Lavaca Bay near the industrial and superfund sites rather than Matagorda Bay? If so, are the benthos being affected more near the contaminated sites, or is the effect constant throughout the estuary? The approach to answer these questions is to use the sediment quality triad (SQT), which is commonly used to assess ecological health of estuaries (Chapman 1990; Chapman et al.^1987^; Chapman and Wang 2001; Hyland et al. 2000). The three SQT components are as follows: (1) measures of chemical contaminants to indicate dose, (2) sediment toxicity tests to measure biological effects at the exposure levels, and (3) benthic community structure data to measure ecological effects in the environment and indicate community status. The three components of the SQT are integrated using multivariate analyses to classify samples and form a “weight-of-evidence” assessment of sediment quality (Long et al. 2003). The overall goal of the present study is to determine which parts of the estuary are being most affected by legacy pollutants and are affecting ecosystem health across the entire MBS.

## Materials and methods

### Study sites

**Fig. 1:**
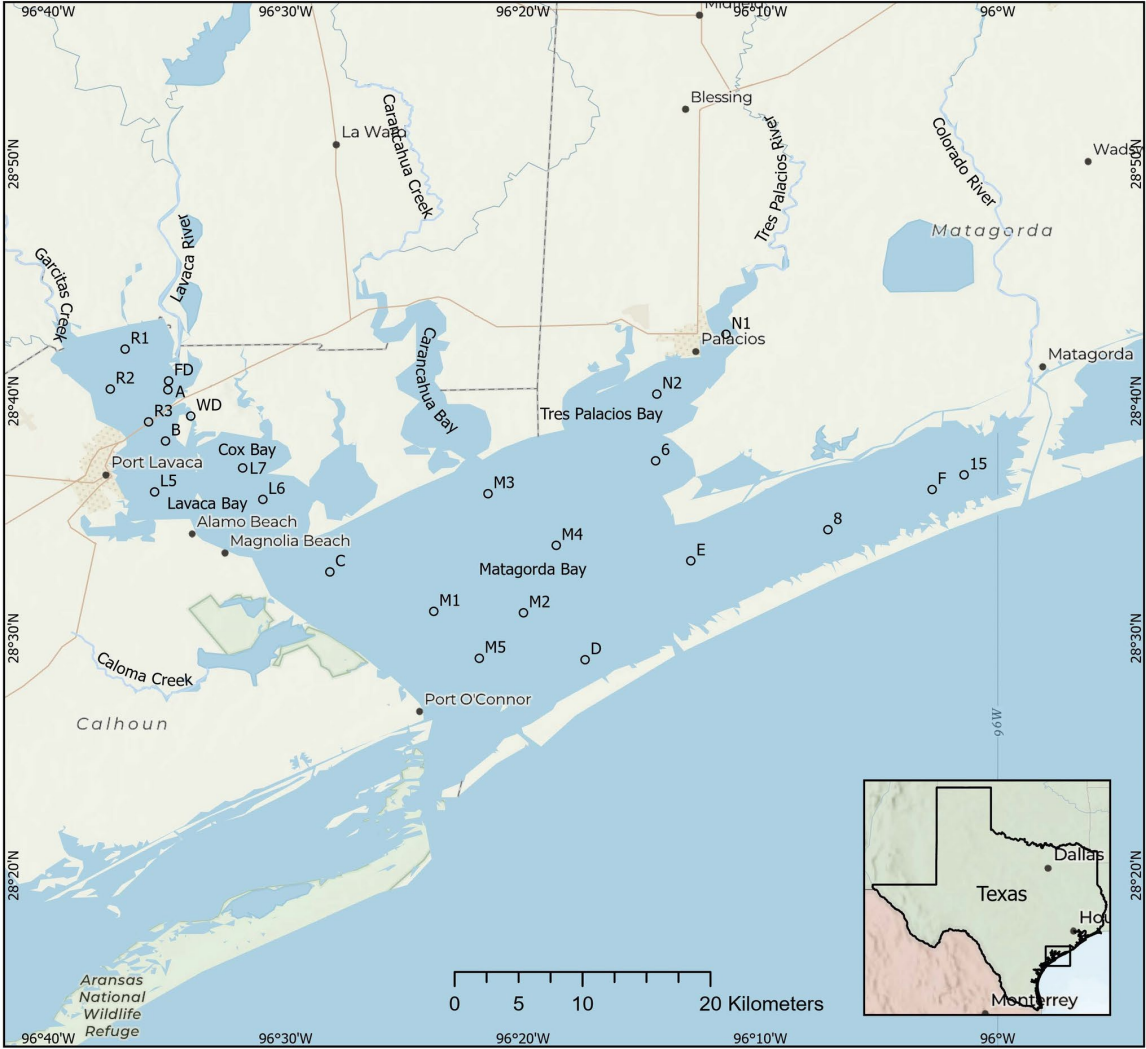
Map of Matagorda Bay system (also known as the Lavaca Colorado Estuary) with stations, river, and county locations. Average location of all stations is 28.608, −96.391 (Table S1 for locations)

Twenty-four stations were sampled during May 2022 (Fig. 1). Seventeen of the stations were chosen because they had been sampled in previous long-term studies conducted by the Harte Research Institute for the Lavaca-Colorado Estuary (LC), the Formosa Plastics Corporation monitoring study, and they were supplemented by an additional seven stations in Lavaca Bay and Matagorda Bay (Harris 2023; Montagna et al. 2025; Table S1). At each station, a Hydrolab multi-parameter sonde was lowered into the water and temperature (± 0.15 ˚C), pH (± 0.1 units), dissolved oxygen (± 0.2 mg l^−1^), water depth (± 0.1 m), and salinity (practical salinity units, psu) were read from the digital display.

### Sediment collection

A hand-held core, 20 cm in length and 6.715 cm diameter, covering an area of 35.4 cm^2^ was used to collect sediment for separate benthos, sediment, and chemical samples. The core tube was securely attached to a PVC pole, allowing deployment over the side of the boat to the desired sediment depth. Upon retrieval, a rubber stopper was immediately placed at the bottom of the core. The core was extruded and then sliced into two depth sections: 0–3 cm deep for chemical, benthic community structure and grain size analyses; and 3–10 cm deep for further analysis of benthic community structure and diversity at depth. The samples were each preserved separately: benthic samples were preserved in formalin, samples for sediment grain size analysis were refrigerated, and samples for chemical contaminant analyses were frozen. In between samples, core tubes were rinsed with acetone using a 500-mL fluorinated ethylene propylene (FEP) squirt bottle, making sure to cover all areas of the cores (Pisarski et al. 2021). Cores were rinsed thoroughly with distilled water, then set in a clean bucket to air-dry until the next set of samples. Three replicates were collected from each station.

A second hand-held core, 20 cm in length and 10 cm diameter (78.54 cm^2^), was used to collect the sediment for toxicological exposures. The sediment was extruded to collect only the surface 0—3 cm depth. A total of 12 cores were collected and 2827 cm^3^ of sediment was placed in 4-L polypropylene wide mouth jars. The toxicology samples were kept refrigerated up to 30 days prior to exposure tests.

### Chemical contaminant assessment

Sediments were analyzed for aliphatic hydrocarbons (HC), total petroleum hydrocarbons (TPH), polycyclic aromatic hydrocarbons (PAH), organochlorine pesticides (OCl’s), polychlorinated biphenyls (PCBs) using an automated extraction apparatus and chromatographic procedures (B&B Laboratories, Inc., College Station, Texas). Total organic carbon (TOC) was measured in oven-dried samples and combusted at 1,350 °C. Trace elements analyzed were arsenic (As), cadmium (Cd), chromium (Cr), lead (Pb), and mercury (Hg) using EPA 6020 A & EPA 7471 by ALS Environmental (Kelso, Washington). Grain size analysis was performed using the wet sieve method by Azimuth Geo Services (Fairfield Bay, Arkansas). The quality assurance/quality control (QA/QC) procedure for the sediment samples included the analyses of a method blank, blank spike, matrix spike/matrix spike duplicate, laboratory duplicate, and applicable reference materials per analytical batch. The raw data and detailed descriptions of all analytical methods and QA/QC procedures are available online in a file named “Technical Report 22–4291 Rev 0.pdf” at 10.7266/9syzmzrd.

Contamination was assessed by comparing probable effects limit (PEL), effects range median (ERM), threshold effects level (TEL), effects range low (ERL), and NOAA screening reference tables to concentrations of inorganic and organic contaminants in samples (Burton 1991; Buchman 2008). PEL is the concentration at which a defined percentage of the benthic population has a toxic response. ERM reflects the 50th percentile of data and represents the levels above which effects are expected to occur. TEL is the concentration at which a toxic response is starting to be observed in the test organisms. ERL reflects the data that is represented in the lower 10th percentile of the effects of each chemical that is identified. Concentrations that are usually below the ERL are expected to rarely affect organisms.

### Toxicity tests

The sediment samples were sent to the NOAA NCCOS Ecotoxicology Branch (Charleston, SC) for toxicity testing where they were held under refrigeration at 4 °C for no more than 30 days until analysis. Three estuarine species were assessed: the grass shrimp (*Palaemon pugio)*, the amphipod (*Leptocheirus plumulosus*), and the polychaete (*Neanthes arenaceodentata*)*.* A 10-day acute sediment toxicity test was followed for *L. plumulosus* and *N. arenaceodentata*. An additional standard toxicity test, The Microtox® assay was also conducted using whole sediment.

Ovigerous grass shrimp were collected in the field (Leadenwah Creek, SC) and held in the laboratory to obtain larval shrimp. The larval shrimp (24–48 h old) were assessed using the sediment elutriate method (Haring et al. 2010). *P. pugio* exposures were 96 h tests, with renewal at 48 h, with three replicates for each site, and ten shrimp in each replicate. The test conditions were: 28 psu, 25 °C, 16 L:8 D photoperiod, 48-h renewal. Sediment samples were stirred in the 4 L jars until homogenized and then placed on a roller for 10 min. 200 mL of sediment was then removed, placed in a 1000 mL beaker and mixed with 800 mL of 28 psu seawater and placed on an orbital shaker table for 60 min (100 rpm). Afterwards, the samples were centrifuged at 3000 rpm for 20 min, to separate the elutriate from the sediment. 200 mL of elutriate was placed into each 500-mL test jar. Water quality (pH, salinity, dissolved oxygen (DO), and temperature) was measured from one replicate before the larval shrimp were put in and then every 24 h. At 48 h the water was renewed with fresh elutriate and mortality was assessed at 96 h when the test ended.

The *L. plumulosus* were shipped from Aquatic Biosystems Inc. and* N. arenaceodentata* were shipped from Aquatic Toxicology Support. Both species were allowed 72 h to acclimate to laboratory conditions prior to testing. Whole sediment tests were conducted with juvenile amphipods, (body length 2–4 mm) and juvenile polychaetes (2–3 weeks old and body length 10–15 mm) (Farrar and Bridges 2011; Johns et al. 1991). The amphipod assay used five replicates per station with 20 amphipods in each replicate and the polychaete assay used five replicates with 5 polychaetes/replicate. Sediment samples were stirred in the 4-L jars until homogenized and then placed on a roller for 10 min. A 175 g aliquot of sediment was added to each 1000-mL beaker. The beaker was then filled with 800 mL (amphipods) and 350 mL (polychaetes) of 28 psu seawater, using a petri dish as a baffle. The beaker was placed in the incubator for 24 h, covered, and aerated. The test conditions were 28 psu, 25 °C, 16 L:8 D photoperiod for amphipods (U.S. EPA 1996) and 28 psu, 20 °C, 16 L:8 D photoperiod for polychaetes (ASTM E-1611 2013). After 24 h, the amphipods and polychaetes were placed in their respective beakers, and water quality was measured daily from two randomly chosen replicates at each station. Ammonia levels were measured using a HACH DR3900 VIS spectrophotometer on Day 0, 2, and 8 (U.S. EPA 1993). On Day 10, samples were sieved, and survival assessed.

### Microtox® solid phase test

Microtox assays were conducted according to the standardized solid phase protocols with the Microtox Model 500 analyzer (Modern Waters Inc., Newark, DE). Sediment was homogenized and a 7.0-g to 7.1-g sediment sample was used to make a series of sediment dilutions with 3.5% NaCl (sodium chloride) diluent. Test samples were placed in a 15 °C water bath for 10 min incubation. Luminescent bacteria (*Vibrio fisheri*) were added to the test concentrations for 20 min incubation. At the end of the incubation period, a column filter was used to separate the liquid phase from the sediment phase, and bacterial post-exposure light output was measured using Microtox Omni Software. An EC50 (the sediment concentration that reduces light output of luminescent bacteria by 50% relative to the controls) value was calculated for each sample in triplicate. Lower EC50 values represent greater sediment toxicity. Due to the high silt–clay content in many of the study sites, we chose to apply the criteria of EC50 < 0.2% and Silt + Clay ≥ 20% to designate toxicity (Ringwood et al. 1997; Table S2).

### Benthic ecological response

The samples for macrobenthos were preserved in formalin, extracted from sediment using a 0.5-mm mesh sieve, and sorted via microscopy. Benthos were enumerated using a dissecting microscope and identified to the lowest taxonomic level possible. For biomass, organisms were dried at 50 °C for 24 h and weighed to the nearest 0.01 mg. For mollusks, the tissue was removed from shells before weighing, and shell lengths were measured.

Species diversity was calculated by replicate and by pooling all three replicate cores for each site. Diversity was calculated using Hill’s diversity number 1 (N1), which is a measure of the effective number of species in a sample and indicates the number of abundant species (Ludwig and Reynolds 1988). It is calculated as the exponentiated form of Shannon diversity index (H′). As diversity decreases N1 will tend toward 1. The Shannon index is the average uncertainty per species in an infinite community made up of species with known proportional abundances (Shannon and Weaver 1949; Hutcheson 1970). Hill’s N1 is used in most analyses because it is easier to interpret than H′. Pielou’s evenness index (J′) represents equitability, expressing how evenly the individuals are distributed among different species. Additional indices calculated include Margalef richness (d), Simpson evenness (1-λ′), average taxonomic diversity (Δ), and average taxonomic distinctness (Δ*) (Warwick and Clarke 1995). All equations for benthic diversity analyses are found in supplementary methods.

Benthic indices to assess sediment health or quality have been developed for freshwater (McPherson et al. 2008), estuarine (Dauer 1993), and coastal habitats (Ritter et al. 2012). All are based on the succession model where good ecological health or high quality is represented by high diversity, *k*-selected, and prominent deep-dwelling organisms and stressed (i.e., polluted environments) are less diverse communities that are dominated by small but numerous *r*-selected species (Pearson and Rosenberg 1976). Two benthic community stress indices were calculated: ABC (Abundance-Biomass-Curves; Clarke and Green 1988; Clarke and Gorley 2015) and AMBI (AZTI’s Marine Biotic Index; Borja et al. 2000, 2019).

### Statistical analyses

Macrofauna community structure was analyzed using non-metric multidimensional scaling (nMDS) in Primer-e software (Clarke and Gorley 2015). The nMDS is based on ordination of Brey-Curtis similarities of square root transformed abundances among stations.

The most toxic chemical components based on sediment quality guidelines reviewed in Burton (2002) were chosen for a multivariate analysis. The trace elements included arsenic (As), cadmium (Cd), chromium (Cr), lead (Pb), and mercury (Hg). Organic compounds included total petroleum hydrocarbons (TPH), alkanes, polycyclic aromatic hydrocarbons (PAH), dichlorodiphenyltrichloroethanes (DDT), and polychlorinated biphenyls (PCB). The sediment characteristics included sand, mud (silt clay), and total organic carbon (TOC). The sediment chemistry data were standardized to a normal distribution with a mean of zero and standard deviation of one and then reduced to principal components (PCs) using principal components analysis (PCA). The four toxicity measurements were also subject to PCA. Because sediment texture affects toxicity, especially the Microtox measure, mud (i.e., silt + clay) content was also included in the PCA.

### SQT analyses

The SQT concept is designed to integrate biological and ecological responses to the environmental setting as characterized by the quantity of sediment contaminants. The statistical approach is based on the concept that the experiment-wide error rate must be controlled and the easiest way to do this is to reduce the number of variables in the analysis (Carr et al. 2000; Long et al. 2003). The integration was performed using a parametric method and a non-parametric method.

The PCA was used as a parametric variable reduction technique for each of the three data sets (chemical, toxicity, and benthic analysis) as described above. This approach acknowledges the multi-dimensional structure of SQT, which was named the M-Triad approach by Fonseca et al. (2021). Spearman correlation coefficients were calculated among the three reduced PCA variables representing the chemical, biological, and ecological data sets.

Ranks of chemical, diversity, and toxicity responses were used in the non-parametric approach. For sediment chemistry, the toxic chemicals As, Cd, Cr, Hg, Pb, total alkanes, total PAH, total TPH, total DDT, and total PCB were used. For diversity, S, H', N1 and Δ was used. For toxicity, the survival of the three species and the concentration of Microtox EC50 were used. The average measurement for every variable at each station was ranked from 1 to 24, where 1 represented the highest chemical concentration, or lowest Microtox EC50 concentration, and lowest diversity or species survival: and 24 represented lowest chemical concentration or highest Microtox EC50 concentration and highest diversity or species survival. The ranks for each variable were averaged by station, and the average ranks were ranked from 1 to 24. The average ranked ranks were converted to percentiles. The percentiles were binned in quartile ranges: 0%–25% for most degraded, lowest diversity, or highest toxicity; 25%–75% is average conditions; and > 75% is most healthy stations with greatest metrics (i.e., highest diversity and lowest toxicity and contaminant concentrations).

A second non-parametric approach is the biota and/or environment matching (BIOENV) method implemented in Primer-e software. The benthic community structure Brey-Curtis similarities were matched with chemical concentrations of As, Cd, Cr, Hg, Pb, total alkanes, total PAH, total TPH, total DDT, and total PCB that were log-transformed and standardized. Thus, the same datasets that were used for PCA were used for BIOENV. BIOENV matches combinations of environmental variables to community structure to determine which abiotic variables best fit faunal patterns.

Results of the SQT analyses were summarized and interpreted using the approach created by Chapman (1990). All of the statistical analyses described above compare relative differences among stations. So, station responses were also compared to absolute values of sediment quality guideline thresholds. The sediment chemistry thresholds were based on Burton (2002) and Buchman (2008) (Table 1). The toxicity thresholds for *L. plumulosus* and *N. arenaceodentata* were based on Bay et al. (2007) (Table S3). The benthic response thresholds were based on ABC (Clarke and Green 1988) and AMBI (Borja et al. 2000) (Table S4).

**Table 1:** Probable effects level (PEL), effects range median (ERM), threshold effects level (TEL), and effects range low (ERL) values (Burton 1991; Buchman 2008). Abbreviations and units: PAH = Polycyclic aromatic hydrocarbons (µg/kg), Trace Elements (mg/kg)

Chemical	PEL	ERM	TEL	ERL	Stations > PEL	Stations > TEL	Stations > ERL
Dibenzo(a,h)anthracene (PAH)	135	260	6.22	63.4		WD	WD
Arsenic	41.6	70	7.24	8.2		6, E, M1, M2, M3, M4, R1	6, E, M3, R1
Cadmium	4.21	9.6	0.68	1.2		6, E, M3, R1	6, E, M3, R1
Chromium	160	370	52.3	81			
Copper	108	270	18.7	34		6, E, M3	
Lead	112	218	30.2	46.7		6, E, M3	
Mercury	0.7	0.71	0.13	0.15		6, B, D, M2, R1, WD	B, D, M2, WD
Nickel	42.8	51.6	15.9	20.9		6, 8, D, E, M3, R1	6, E, M3
Silver	1.77	3.7	0.73	1.0	6, E, M3	6, E, M3, R1	6, E, M3, R1

## Results

### Physical factors

The water temperatures ranged from 27.34 to 28.73 °C among stations with cooler temperatures in the deepest part of the bay (Fig. S5). The maximum water depth was 4.1 m with shallower depths near river mouths and deeper depths in the center of Matagorda Bay (Fig. S5). Station WD in Lavaca Bay was deep because it is in the Alcoa dock area. Salinity ranged from 26.17 to 29.88 psu with the lowest salinities in Lavaca Bay near the Lavaca River mouth (Fig. S6). The dissolved oxygen ranged from 5.84 to 8.84 mg/L (Fig. S7). The sediment at half of the sites in Upper Lavaca, Lower Lavaca, and Tres Palacios Bays (15, A, B, F, FD, L6, L7, M5, N1, N2, R2, and R3) was composed of mostly sand, and the other half, mostly in Matagorda Bay, was composed of mud, i.e., silt + clay.

### Chemical factors

A summary of sediment threshold levels of PAH, PCB, TPH, TOC, OCl, and trace elements is presented in (Table 1). There were no elevated levels of PAH, PCB, TPH, TOC, or OC at any of the 24 stations. Concentrations of the organic pollutants were below effects range median (ERM) and probable effects level (PEL) thresholds at all stations, with the exception of one PAH (dibenzo(ah)anthracene) that was above effects levels located at the Witco discharge (WD) station in lower Lavaca Bay.

There were elevated levels of seven of the nine trace elements (arsenic, cadmium, copper, lead, mercury, nickel, and silver) mostly located in the lower part of Lavaca Bay (Table 1). Chromium and zinc had concentrations below all targeted bioeffect guidelines. Silver was the only metal with concentrations above the upper-threshold ERM or PEL guideline values, which only occurred at stations 6, E, and M3. Four stations, located at the eastern side of Matagorda Bay (6, 8, E, M3) had elevated levels in excess of the lower threshold ERL or TEL values for all the above seven metals. Four stations located in the central basin of Matagorda Bay (M1, M2, M3, M4) had elevated levels of arsenic above ERL or TEL guidelines. Station R1 had elevated levels of arsenic, cadmium, and nickel. There were six stations that had elevated levels of mercury (6, B, D, M2, R1, and WD, Table 1).

**Fig. 2:**
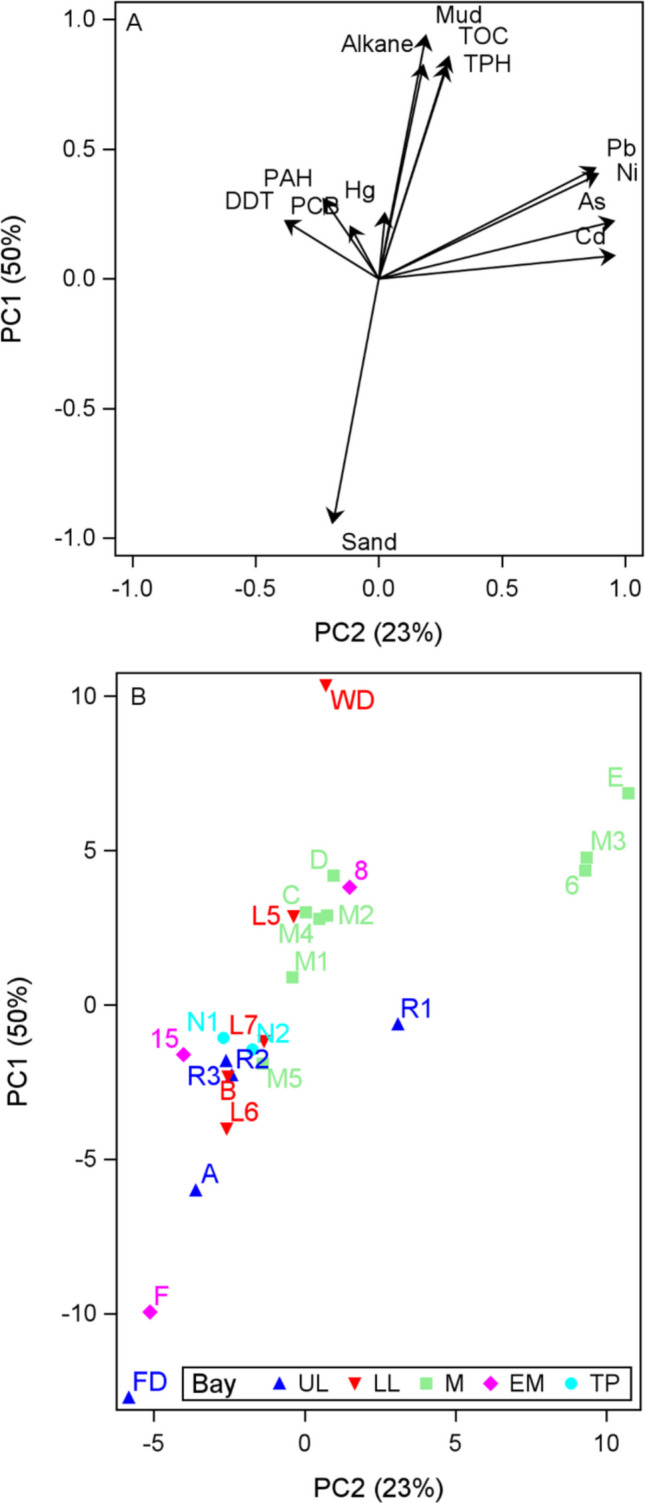
Principal component (PC) and percent variance explained by sediment characteristics and chemical measurements. **A** Rotated variable loads for PC1 versus PC2. Abbreviations: Mud = silt + clay, As = Arsenic, Cd = Cadmium, Cr = Chromium, Cu = Copper, Pb = Lead, Hg = Mercury, Ni = Nickel, Ag = Silver, Zn = Zinc, DDT = dichlorodiphenyltrichloroethanes, PAH = polycyclic aromatic hydrocarbons, PCB = polychlorinated biphenyl, and TPH = total petroleum hydrocarbons. **B** Station sample scores. Data labels are station names. Color and symbol codes: blue triangle = Upper Lavaca Bay, magenta down triangle = Lower Lavaca Bay, green square = Matagorda Bay, pink diamond = eastern arm of Matagorda Bay, light blue circle = Tres Palacios Bay

Three principal components (PC) were extracted from the chemistry and grain size mean at each station (Fig. S8). PC1 represents 50% of the variance explained because of the variability among stations with high concentrations of alkanes, TPH, TOC, and mud (silt + clay), which is the inverse of sandier stations (Fig. 2A).Thus, PC1 represents a new variable describing the distribution of sediments with high concentrations of hydrocarbons and mud versus sandier sediments with low concentrations. PC2 represents 23% of the variance explained with an inverse relationship between trace elements above threshold limits and chlorinated hydrocarbons (DDT and PCB) and PAH. PC3 represents 12% of extracted variance and is an indicator of synthetic contaminants because PCB and DDT were inversely related to each other (Fig. S8).

The sample score for station WD is highest for PC1 and lowest for station FD (Fig. 2B). The PC2 sample scores for stations 6, E, and M3 are highest because these three stations had the highest trace element concentrations (Figs. S9). These stations had the highest elemental concentrations of all the Matagorda Bay stations. Stations with high concentrations of DDT were found in East Matagorda, Tres Palacios, and Upper Lavaca bays all near creek or river mouths. The stations with high concentrations of PAH, PCBs, and mercury were frequently found at stations in Matagorda Bay and Lower Lavaca Bay.

### Toxicity tests

**Fig. 3:**
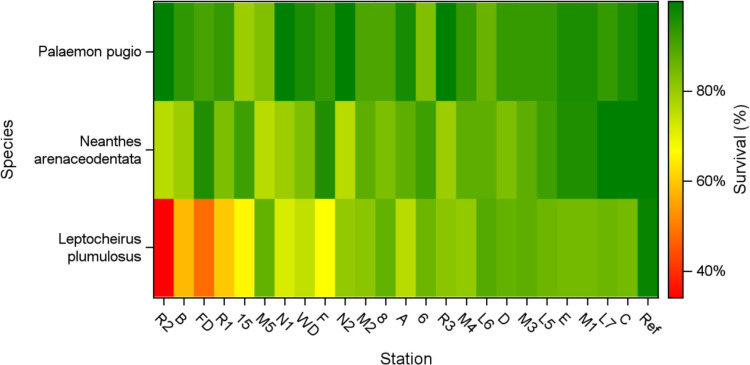
Heat map of average percent survival for three species. Stations ordered by average survival of all three species. Reference (Ref) site is Leadenwah Creek, South Carolina, USA

The three organisms evaluated had different survival patterns when exposed to sediment from stations near river inlets compared to the primary bay (Fig. 3). Overall, *P. pugio* had the highest survival rate, *N. arenaceodentata* had moderate survival, and *L. plumulosus* had the lowest survival, especially at stations in the Upper Lavaca Bay (stations R1, R2, and FD) and Upper East Matagorda Bay (Station 15). All three species had highest survival exposed to the reference (Ref) sediment.

Upper Lavaca Bay had the lowest survival rates (< 80%) and the highest survival (> 80%) was in Matagorda Bay (Fig. 3). Station R2, which is directly across from the industrial plants and in front of Placedo Creek and Garcitas Creek, had the lowest survival rate overall (40%) in spite of high survival for *P. pugio*. The highest survival rate (100% survivability) was at station C, which is at the edge of the lower Lavaca Bay and beginning of the Matagorda Bay.

For Microtox® sediment toxicity, the silty sediment has very low EC50 values (indicating toxicity), whereas sandy sediments had very high EC50 values (indicating not toxic) (Table 2). Four stations (8, 15, L6, and N2) exhibiting toxicity were near creek or river mouths, indicating there is an influence of creeks or rivers on the microbial toxicity. But six stations (6, D, E, M1, M2, and M3) were in Matagorda Bay where sediments are fine (≥ 85% silt + clay).

**Table 2:** Mean percent effect concentration causing 50% survival (EC50) in the Microtox test and percent silt + clay. Stations classified as toxic if EC50 ≤ 0.2% and silt + clay ≥ 20% (Ringwood et al. 1997). Bay abbreviations: EMatagorda = Eastern arm of Matagorda, UpLavaca = Upper Lavaca, LoLavaca = Lower Lavaca

Station	Bay	Mean EC50 (%)	% Silt + Clay	Classification
6	Matagorda	0.102	85.3	Toxic
8	EMatagorda	0.089	93.0	Toxic
15	EMatagorda	0.132	86.0	Toxic
A	UpLavaca	0.667	41.6	Not toxic
B	LoLavaca	0.477	69.8	Not toxic
C	Matagorda	0.213	95.1	Not toxic
D	Matagorda	0.149	88.3	Toxic
E	Matagorda	0.082	94.4	Toxic
F	EMatagorda	0.278	37.3	Not toxic
FD	UpLavaca	1.276	17.9	Not toxic
L5	LoLavaca	0.339	93.4	Not toxic
L6	LoLavaca	0.194	67.4	Toxic
L7	Matagorda	0.316	82.1	Not toxic
M1	Matagorda	0.149	89.0	Toxic
M2	Matagorda	0.133	93.5	Toxic
M3	Matagorda	0.166	91.6	Toxic
M4	Matagorda	0.205	89.8	Not toxic
M5	Matagorda	0.254	73.2	Not toxic
N1	TresPalacios	0.591	73.6	Not toxic
N2	TresPalacios	0.199	64.1	Toxic
R1	UpLavaca	0.428	68.5	Not toxic
R2	UpLavaca	0.391	67.4	Not toxic
R3	UpLavaca	0.415	74.8	Not toxic
WD	LoLavaca	0.207	99.7	Not toxic

**Fig. 4:**
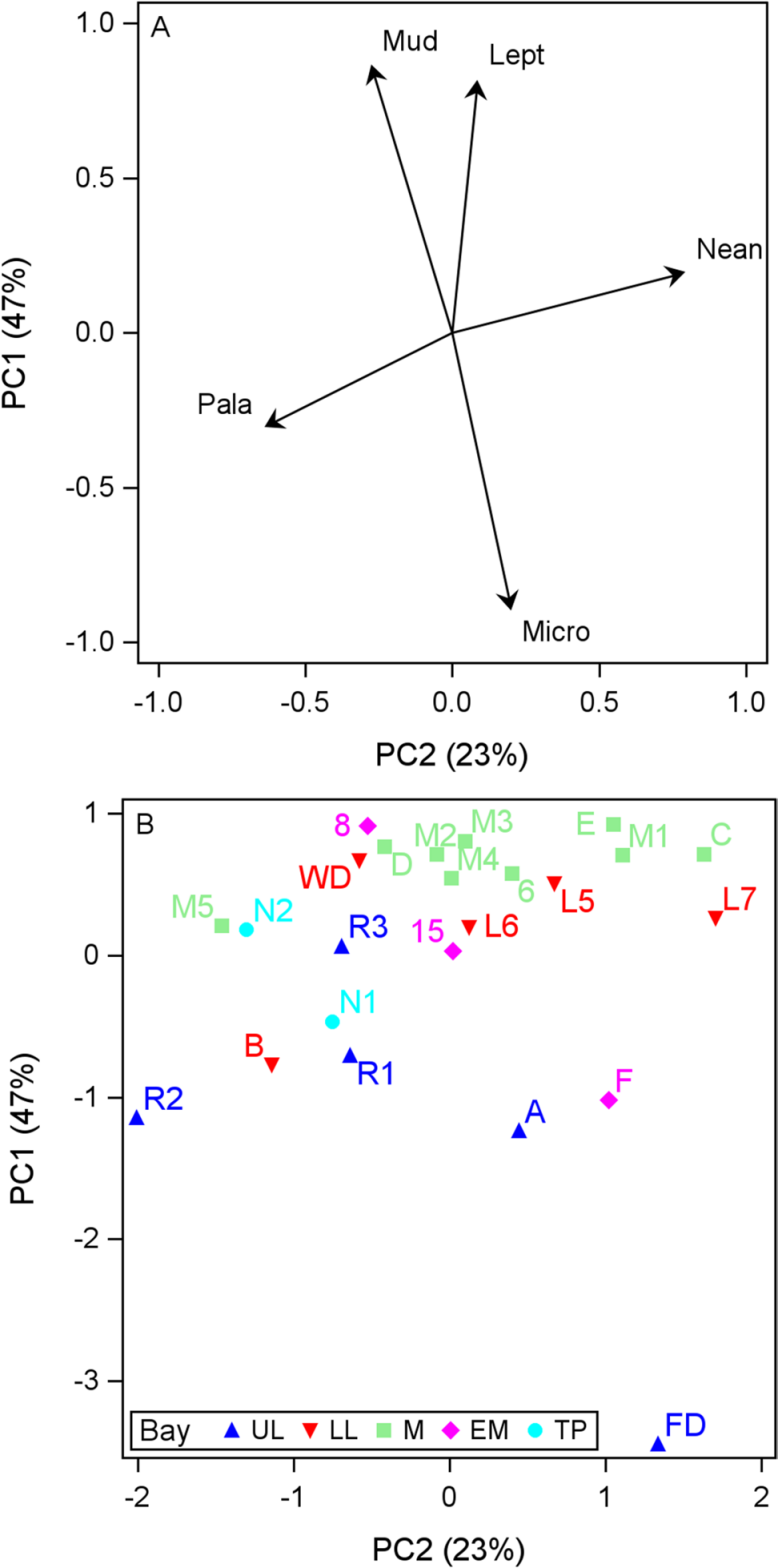
Principal component (PC) analysis of toxicity as indicated by survival of three species or Microtox (i.e., the concentration of sediment causing a reduction in bacterial fluorescence). **A** Variable loads. Abbreviations: Lept = *Leptocheirus plumulosus*, Nean = *Neanthes arenaceodentata*, Pala = *Palaemon pugio*, and Micro = Microtox. **B** Station sample scores. Data labels are station names. Color and symbol codes: blue triangle = Upper Lavaca Bay, magenta down triangle = Lower Lavaca Bay, green square = Matagorda Bay, pink diamond = eastern arm of Matagorda Bay, light blue circle = Tres Palacios Bay

All toxicity tests were compared in a PCA (Fig. 4). The first component of PCA (PC1) explains 47% of the variability, and it represents high mud (silt + clay) content and *L. plumulosus* survival, which is inversely correlated with low Microtox concentration. Thus, high mud content is associated with low amphipod toxicity and high bacteria toxicity (i.e., microbe exposure to low concentrations of sediment). The second component, PC2, explains 23% of the variability and represents opposite trends of high polychaete survival inversely correlated to low shrimp survival. The high mud content and high amphipod survival occurred mostly in Matagorda Bay (Fig. 4B) while the high toxicity occurred mostly in upper Lavaca Bay and the eastern arm of Matagorda Bay.

### Benthic diversity

A total of 47 species were found among the 72 samples from 24 stations (Table S5). *Mediomastus ambiseta* was the dominant species (468 total estuary-wide) found in all stations, except M2, with a mean abundance 1847.60 n/m^2^. The second most dominant species was *Paraprionospio pinnata* with a total of 37 individuals estuary wide (mean abundance 145.76 n/m^2^) and was found in 16 out of 24 stations mostly in Upper and Lower Lavaca Bay and Tres Palacios Bay. The most common class of organisms was Polychaeta, accounting for a little over half of the organisms collected. Station D had the highest species richness with 15 species, and L5 had the lowest with only two species, *Mediomastus ambiseta* and *Macoma mitchelli*. Station15, located in eastern Matagorda Bay, had the greatest number of *Mediomastus ambiseta*. The stations that had lowest abundance were in Upper Lavaca Bay and Matagorda Bay.

**Fig. 5:**
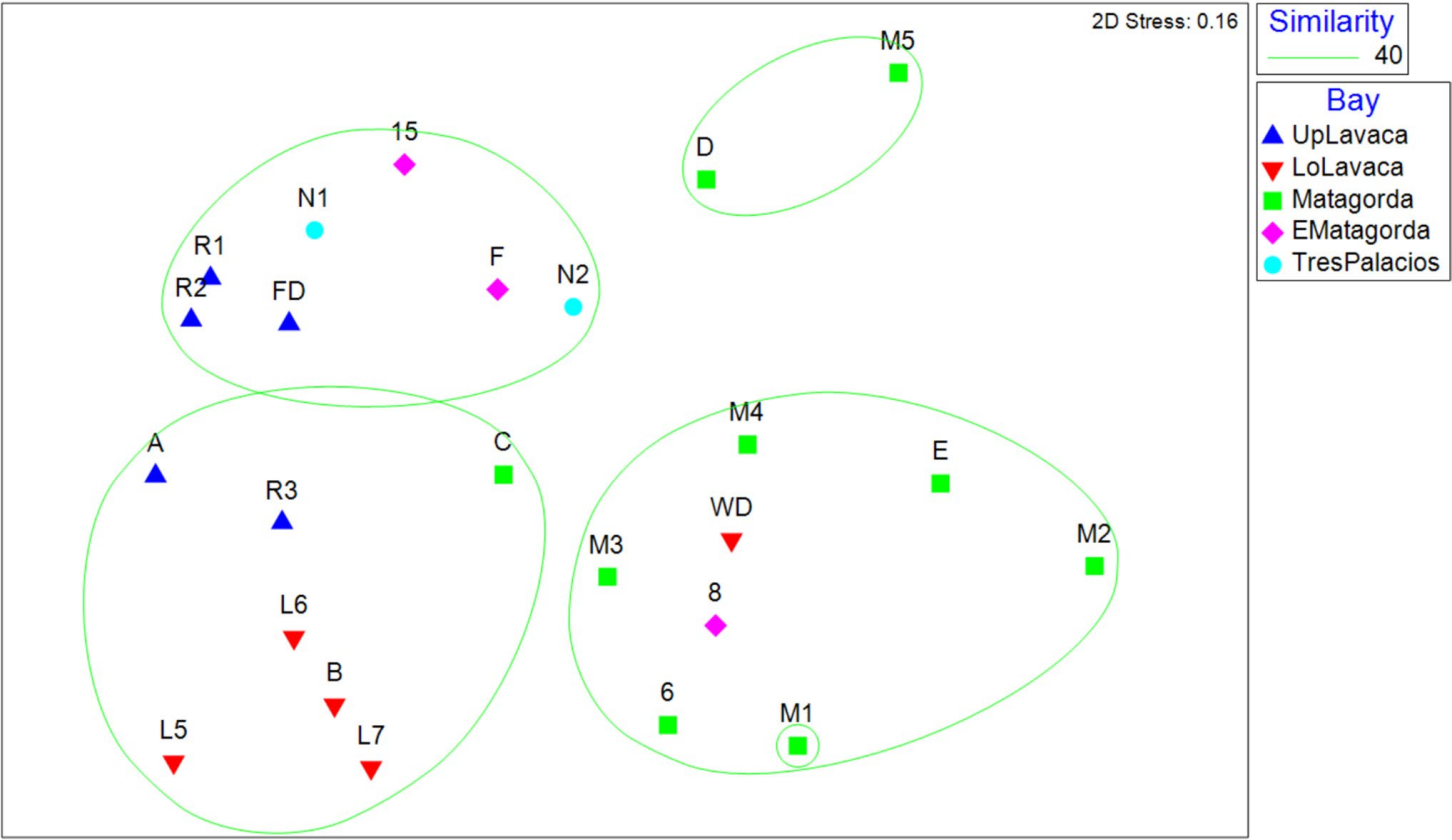
Similarity of benthic community structure among stations based on non-metric multidimensional scaling and cluster analysis. Symbols are bays within the Lavaca-Colorado Estuary. Bay region abbreviations: EMatagorda = Eastern arm of Matagorda, UpLavaca = Upper Lavaca, LoLavaca = Lower Lavaca

The benthic community structure composition was similar within bays (Fig. 5). Station WD (Witco Discharge site) is an exception because it was mostly related to stations in Matagorda Bay. Stations R1, R2, and FD (Upper Lavaca Bay) were similar to stations in eastern Matagorda Bay and Tres Palacios Bay. Stations near the Lavaca River were similar in benthic community structure to stations near the Colorado River and Tres Palacios River (Fig. 5, left top similarity group).

The best matching of benthic community structure with environmental variables was a correlation of 0.163 for Cd and Ni (Table S6).

The three components of diversity (richness, evenness, and diversity) were calculated for each station (Table 3). Richness (S) was very low with the highest number of species found in station D (15 species). Yet, Pielou evenness (J′) and Shannon diversity (H′) were above 0.05 at all stations. Evenness and diversity compared to richness had coefficients of less than 0.05 meaning that there was little to no correlation between the number of species and how evenly they were distributed around the bay.

**Table 3:** Average benthic macrofauna community metrics. Abbreviations: N = abundance extrapolated to n/m^2^, S = Richness number of species, d = Margalef Richness, J′ = Pielou evenness, H′ = Shannon diversity, 1-λ′ = Simpson evenness, N1 = Hill N1 number of dominant species, Δ = average taxonomic diversity, Δ +  = average taxonomic distinctness, mass = biomass extrapolated to g/m^2^, ABC = abundance-biomass curve stress index. Diversity and evenness indices are per 101 cm^2^

Station	N	S	d	J'	H'	1-λ'	N1	Δ	Δ +	Mass	ABC	AMBI
6	1418	9	1.10	0.95	2.08	0.86	8.03	63.9	74.1	1.252	0.45	2.30
8	2647	8	0.89	0.86	1.78	0.80	5.93	51.3	63.8	1.768	0.15	3.11
15	12,291	10	0.96	0.44	1.01	0.41	2.75	35.0	85.5	0.972	−0.32	4.06
A	2553	5	0.51	0.51	0.83	0.38	2.28	33.6	88.0	2.141	0.13	4.00
B	851	5	0.59	0.89	1.43	0.72	4.17	64.7	90.2	2.624	0.33	3.50
C	2458	12	1.41	0.85	2.10	0.83	8.20	73.5	88.9	1.492	0.19	2.94
D	6807	15	1.59	0.71	1.92	0.74	6.83	61.0	82.2	6.955	0.28	3.21
E	756	5	0.60	0.97	1.56	0.78	4.76	59.5	76.0	0.107	0.72	2.25
F	5200	7	0.70	0.50	0.97	0.43	2.63	31.3	72.9	0.494	−0.19	4.23
FD	5862	6	0.58	0.47	0.83	0.37	2.30	31.9	87.2	5.570	0.04	4.04
L5	567	2	0.16	0.65	0.45	0.28	1.57	27.8	100.0	0.482	0.25	4.00
L6	1040	4	0.43	0.84	1.16	0.65	3.20	47.4	73.5	0.100	0.10	3.00
L7	662	7	0.92	1.00	1.95	0.86	7.00	78.3	91.3	3.194	0.91	2.36
M1	1796	14	1.73	0.96	2.52	0.91	12.49	75.9	83.5	1.320	0.47	1.82
M2	945	6	0.73	0.90	1.61	0.76	5.00	64.4	84.6	1.332	0.25	2.25
M3	1229	8	0.98	0.91	1.88	0.82	6.59	66.1	80.9	0.993	0.62	3.35
M4	2080	10	1.18	0.92	2.11	0.86	8.28	77.8	90.9	0.746	0.45	3.00
M5	6713	14	1.48	0.70	1.84	0.72	6.31	69.9	96.4	24.141	0.32	1.50
N1	4255	7	0.72	0.67	1.31	0.60	3.70	52.6	87.3	4.314	0.27	3.83
N2	3215	10	1.11	0.85	1.96	0.82	7.12	69.4	84.6	1.971	0.30	3.26
R1	8131	4	0.33	0.42	0.58	0.27	1.78	21.7	80.2	1.224	−0.04	4.34
R2	5767	7	0.69	0.39	0.75	0.32	2.12	27.2	85.3	1.351	−0.09	4.25
R3	1985	7	0.79	0.70	1.36	0.63	3.90	53.2	84.9	0.394	0.08	3.50
WD	945	4	0.44	0.92	1.28	0.70	3.60	50.1	71.4	0.746	0.54	3.90

Each station was categorized based on ABC scores (Table S4). Four stations (15, R1, R2, and F) appeared to be highly stressed, and these stations were located near Garcitas Creek, Lavaca River, and the Colorado River mouths. Station WD (Witco Discharge site) located near the ALCOA Plant, had the highest concentration of chemical contaminants, but was unstressed according to the ABC index. Partially stressed stations (FD, L6 and R3) were also located near creek or river mouths.

**Fig. 6:**
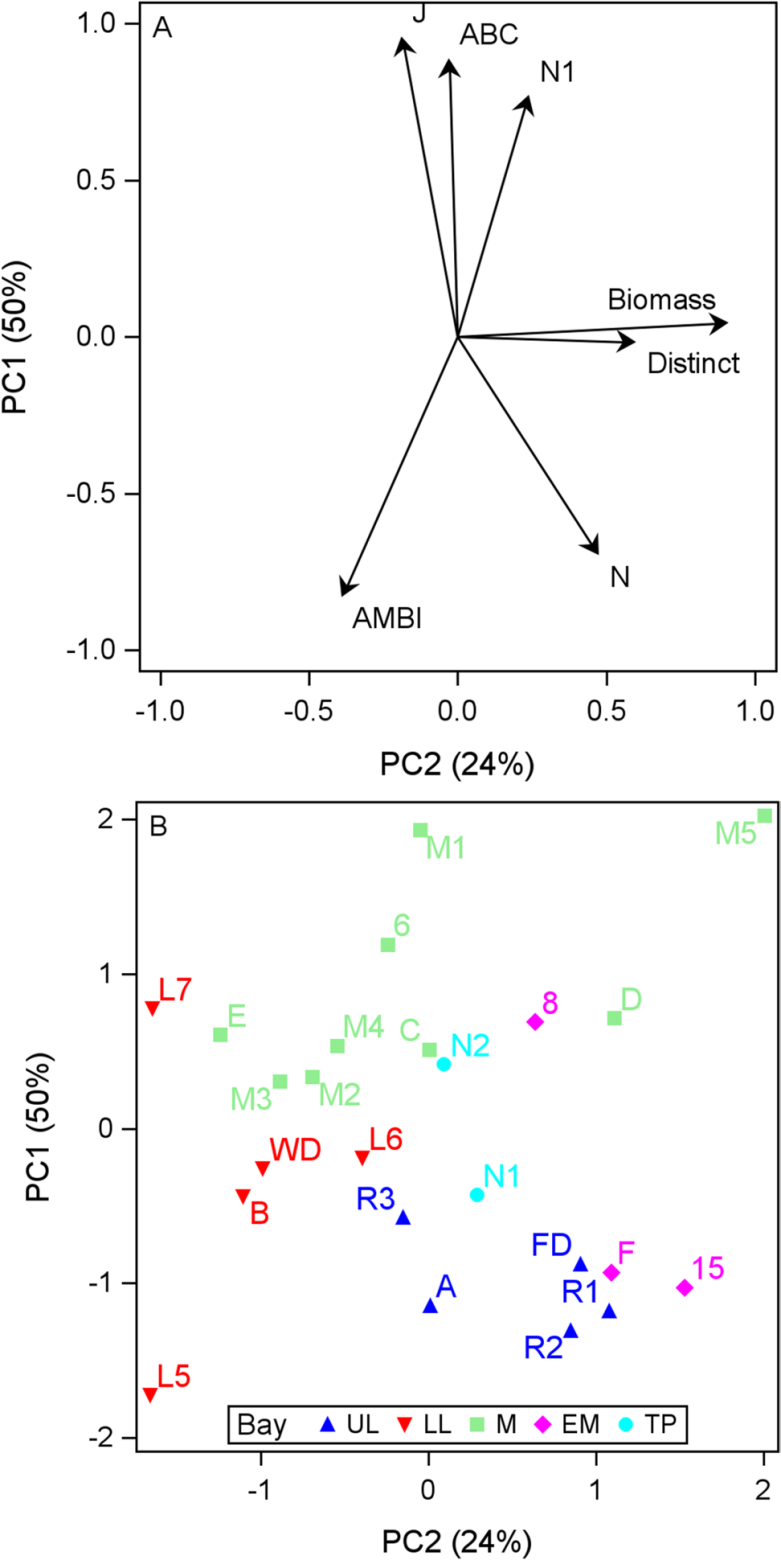
Principal Component (PC) analysis of seven benthic metrics and the percentage of variance explained by the new PC variables. **A** Variable loads. Abbreviations: N = abundance, J = Pielou evenness, N1 = Hill diversity, Distinct = evenness of taxonomic distinctness (Delta +), AMBI index, and the ABC stress index. **B** Station sample scores. Data labels are station names. Color and symbol codes: blue triangle = Upper Lavaca Bay, magenta down triangle = Lower Lavaca Bay, green square = Matagorda Bay, pink diamond = eastern arm of Matagorda Bay, light blue circle = Tres Palacios Bay

A PCA was performed on a subset of diversity metrics (Fig. 6). A subset was chosen to avoid redundancy because many of the diversity metrics are highly correlated with one another (Table S7). The subset included abundance (N), Pielou evenness (J′), Hill diversity (N1), taxonomic diversity (Delta = Δ), evenness of taxonomic distinctness (Distinct = Δ +), Biomass, and the ABC stress index. The PC1 variable explained 52% of the extracted variance and was represented by high values of J, N1, ABC, and Δ, which were inversely correlated with abundance. The PC2 variable explained 23% of the variance and was represented by high values of biomass and evenness of taxonomic distinctness (Δ +). Thus, PC1 represents high diversity and PC2 represents high biomass. High evenness, diversity, and biomass relative to abundance (i.e., ABC index) occurred mostly in Matagorda Bay and Tres Palacios Bay (Fig. 6B). Low abundance and AMBI index values occurred mostly in upper and lower Lavaca Bay and the eastern arm of Matagorda Bay, which all are near river inflow locations.

### SQT analysis: parametric approach

A simple approach to a SQT analysis is to compare the three PCAs for chemistry, toxicity, and macrofauna metrics by correlation among the PC station scores (Table 4). High macrofauna metrics (MacrPC1) were positively correlated with high survival (SurvPC1). High survival (SurvPC1) was positively correlated with high organic content (ChemPC1) and with low organic contaminants (DDT and PAH) and high trace elements (ChemPC2).

**Table 4:** Spearman correlation coefficients (r) comparing three PCAs for macrofauna (i.e., Fig. 6: MacrPCA1 and MacrPCA2), toxicity (i.e., Fig. 4: SurvPC1 and SurvPC2), and chemicals (i.e., Fig. 2: ChemPC1, ChemPC2, and ChemPC3). P is the probability that r = 0 where n = 24 stations

	**MacrPC1**		**MacrPC2**		**SurvPC1**		**SurvPC2**		**ChemPC1**		**ChemPC2**	
	**r**	**P**	**r**	**P**	**r**	**P**	**r**	**P**	**r**	**P**	**r**	**P**
**MacrPC2**	−0.04	0.8559										
**SurvPC1**	0.58	0.0031	−0.31	0.1470								
**SurvPC2**	0.05	0.8087	−0.23	0.2900	0.20	0.3382						
**ChemPC1**	0.31	0.1375	−0.25	0.2329	0.87	< 0.0001	0.09	0.6832				
**ChemPC2**	0.26	0.2247	−0.20	0.3552	0.81	< 0.0001	0.03	0.9005	0.88	< 0.0001		
**ChemPC3**	0.04	0.8623	−0.45	0.0275	0.48	0.0171	−0.01	0.9743	0.58	0.0030	0.58	0.0029

### SQT analysis: non-parametric approach

**Fig. 7:**
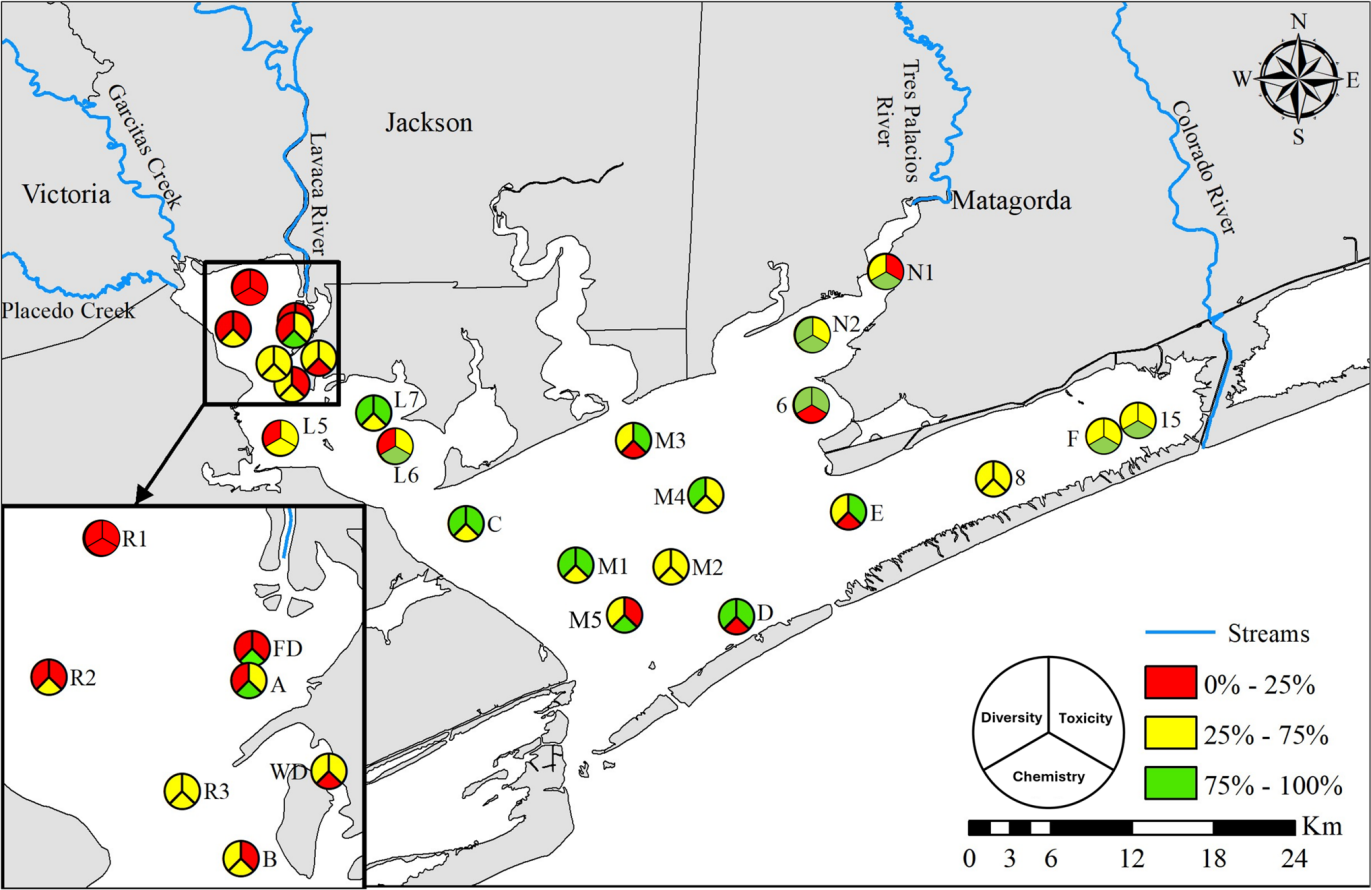
Map of summarized average sediment quality triad quantiles at stations. Ranks based on Table 4

The SQT data are summarized (Fig. 7) for each station based on a ranking system (Table 5). The rank of each component from 0–25% (red) indicates the first quartile of high contamination, toxicity, or low diversity; ranks in the middle quartiles between 25 and 75% (yellow) indicate average contamination, toxicity, or diversity; and ranks in the fourth quartile > 75% (green) indicate low contamination and toxicity, and high benthic diversity. The least contaminated station was FD, in upper Lavaca Bay, and the most contaminated station was WD, in lower Lavaca Bay (Fig. 7, Table 5). Only one station, R1, ranked in the lowest quartile for all three metrics. Four of the Matagorda Bay stations (6, D, E, and M1) were in the lowest quartile for sediment chemistry, meaning they had the highest contamination concentrations among stations. All stations in secondary bay areas near river or creeks mouths had poor to moderate survival and diversity.

**Table 5:** Ranking of stations for the sediment quality triad. Chemistry rank based on average rank of concentrations of As, Cd, Hg, Pb, Ni, total alkanes, total PAH, total TPH, total DDT, and total PCB. Survival rank based on average survival of three species. Diversity rank based on average of ranks of diversity indices: S, H′, N1, and Δ

Station	Diversity		Toxicity		Chemistry	
	**Rank**	**Percentile**	**Rank**	**Percentile**	**Rank**	**Percentile**
6	18	75.0	18	75.0	4	16.7
8	15	62.5	17	70.8	11	45.8
15	9	37.5	7	29.2	21	87.5
A	3.5	14.6	8	33.3	22	91.7
B	13	54.2	1	4.2	16	66.7
C	22	91.7	22	91.7	9	37.5
D	18	75.0	19	79.2	3	12.5
E	11	45.8	24	100.0	2	8.3
F	7	29.2	11	45.8	23	95.8
FD	5	20.8	3	12.5	24	100.0
L5	1	4.2	15	62.5	7	29.2
L6	6	25.0	16	66.7	19	79.2
L7	18	75.0	20	83.3	13	54.2
M1	24	100.0	23	95.8	12	50.0
M2	14	58.3	14	58.3	10	41.7
M3	16	66.7	21	87.5	5	20.8
M4	23	95.8	10	41.7	8	33.3
M5	20	83.3	5	20.8	20	83.3
N1	10	41.7	6	25.0	17	70.8
N2	21	87.5	13	54.2	18	75.0
R1	2	8.3	2	8.3	6	25.0
R2	3.5	14.6	4	16.7	14	58.3
R3	12	50.0	9	37.5	15	62.5
WD	8	33.3	12	50.0	1	4.2

## Discussion

The primary objective was to conduct a sediment quality triad assessment to determine if sediment chemical contamination was present in the Matagorda Bay System (MBS) and if it was at concentrations high enough to cause toxicity or community change in benthic organisms. The results indicate that 17 of the 24 stations had fair to poor survival in sediment toxicity tests (Fig. 3), and 17 out of the 24 stations had fair to low benthic diversity (Table 3). However, there was little correlation between toxicity and high concentrations of sediment contamination. Station FD, at the Formosa Discharge point, was the least contaminated (ranked 24; Upper Lavaca Bay), but was accompanied by high sediment toxicity and poor benthic condition (coded red in Fig. 7), while the most contaminated station WD, Alcoa Witco Discharge site, (ranked 1; Lower Lavaca Bay) was accompanied by only moderate levels of benthic condition and sediment toxicity. Only one station (R1, Upper Lavaca Bay) had all three components of the SQT coded red (high contamination, high toxicity, and low diversity). In general, survival in toxicity tests was positively correlated with diversity (r = 0.53, Table S7), and the ABC index (r = 0.50, Table S7). However, there were no correlations among all stations between sediment chemistry and toxicity or benthic metrics (Table S7).

### Sediment contaminants

Most stations had low to moderate levels of the measured contaminants, but only three of these stations (6, E, and M3) rose above any of the probable effects level (PEL) chemical threshold limits (Table 1). Of the five stations with high sediment contamination (coded red in Fig. 6), one (WD) had intermediate levels of benthic condition or sediment toxicity, and two (6 and D) were accompanied by good benthic condition and low sediment toxicity A total of 46% (i.e., 11) of the stations had chemical contaminant concentrations over TEL and ERL thresholds for seven trace elements (arsenic, cadmium, copper, lead, mercury, nickel, silver) and one PAH (dibenzo (a,h) anthracene).

The concentrations of PAHs found in Lavaca and Matagorda Bays are comparable concentrations found in other nearby Texas bays including Corpus Christi Bay, Aransas Bay, and San Antonio Bay (Lloyd et al. 2024). Lloyd et al. (2024) concluded that PAHs in Texas bays are likely the result of a pyrogenic source, particularly petroleum combustion.

The legacy chemical contaminants found throughout the entire estuary are just one possible explanation for poor sediment quality (indicated by the low benthic diversity and high sediment toxicity) found in many places. However, one limitation is that many potential contaminants were not measured, such as: agricultural pesticides, herbicides, phthalates, dioxins, furans, alkylphenols, pharmaceuticals, perfluoroalkyl and polyfluoroalkyl substances (PFAS), and polybrominated diphenyl ethers (PBDE). It is known that some of these contaminants could be causing benthic degradation (Lee and Khim 2022; Moon et al. 2008; Khim et al. 1999).

In Matagorda Bay, PAHs and PCBs are known to accumulate in gill and mantle tissues of eastern oysters (*Crassostrea virginica*) and muscle and liver tissues of gafftop sail catfish (*Bagre marinus*), red drum (*Sciaenops ocellatus*), and spotted sea trout (*Cynoscion nebulosus*) (Mortuza et al. 2025). But more interesting is that emerging pollutant nano(micro)plastics (NMPs) body burdens were 2000 × –60,000 × higher than PAHs and PCBs. The NMPs were not measured in the current study and could be another important contaminant that was not measured but could explain results in the current study.

### Benthic communities

Benthic community analysis has been used as an ecological indicator in many environmental assessment studies (Reish 1972; Dauer 1993; Pinto et al. 2009). However, estuaries are naturally stressed environments, experiencing dramatic salinity fluctuations, which is known to reduce benthic community diversity (Van Diggelen and Montagna 2016). So, care must be taken when applying benthic indices to areas with salinity gradients (Zettler et al. 2007). Salinity fluctuation was not evident during the current study because salinity ranged from 27–30 during sampling (Fig. S4).

The species (*Mediomastus ambiseta, Capitella capitata, and Steblospio benedicti*) were the most abundant in the Matagorda Bay system (Table S5). *M. ambiseta* and *S. benediciti* are pioneer species that have fast reproduction rates and are known to be tolerant to a highly variable/disturbed environment (Dauer 1993; Kalke and Montagna 1991; Mannino and Montagna 1997). *C. capitata* is also an opportunistic species and is indicative of stressful or polluted waters (Grassle and Grassle 1976; Reish 1971; Tautsumi 1987). These species can indicate a chemically and physically stressed environment, but other factors such as salinity fluctuations, physical disturbances, and nutrient declines must be taken into consideration as well.

### Toxicity analysis

There was also an obvious influence of river discharge on survival and benthic community diversity. Overall, toxicity had little to no relationship to sediment chemistry, but there were a few stations that were more sensitive to contamination than others. The finding that there was no relationship between sediment chemistry and toxicity indicates that contaminants may not be bioavailable, that contaminants causing toxicity were not measured, or the benthic response is due to other stressor (Chapman 1990). For example, factors such as precipitation and local salinity patterns were shown to be strong drivers of benthic diversity in the MBS (Pollack et al. 2011). Low dissolved oxygen concentrations are drivers of benthic abundance, biomass, and diversity along much of the Texas coast, but especially in the MBS (Montagna et al. 2025). Low dissolved oxygen stemming from nutrient and organic matter loadings is a potential cause for degraded benthos in Chesapeake Bay sites that did not correspond with chemical contaminants or toxicity data (McGee et al. 2009). Stations R1, R2, and FD, with the lowest survival were near the Lavaca River mouth in Upper Lavaca Bay, meaning various contaminants could emanate from runoff to the rivers and could be affecting the benthic community (Fig. 5). *P. pugio* had the highest survival rate, but this might be due to the shrimp not being directly exposed to the sediment and only to the elutriate water extracted from the sediment. The sites where both *N. arenaceodentata* and *L. plumulosus* were impacted, but *P. pugio* were not, which indicates the contaminants were not water soluble, and thus not bioavailable to *P. pugio*.

One odd, but interesting, finding is the opposite responses of *L. plumulosus* and Microtox toxicity (Fig. 4). Microbial toxicity was found mostly in Matagorda Bay and amphipod toxicity was found mostly in Lavaca Bay near the Lavaca River mouth and the eastern arm of Matagorda Bay near the Colorado River mouth. Microbial toxicity appears to be related to fine sediments in the open primary bay with high concentrations of metaloids (Fig. 2), but amphipod toxicity appears to be related to coarser sediments with high concentrations of chlorinated hydrocarbons (e.g., DDT or PCB in the secondary bays with more freshwater influence. The amphipod L. *plumulosus* also was the most sensitive species tested. Juvenile *Leptocheirus* have been shown to be sensitive to PAHs, trace metal elements, and pesticides in Chesapeake Bay, USA (McGee et al. 2004). The amphipod toxicity was also linked to trace metal elements, PAHs, and PCBs in Baltimore Harbor, USA (Manyin and Rowe 2006). *Leptocheirus* has also been shown to have good agreement between laboratory toxicity and sediment contamination (McGee et al. 2004),

### Comparison to other sediment quality assessments

The SQT data was summarized based on correlations among multivariate PC scores (Table 4) and ranked quantiles of (Fig. 7). These approaches compare stations to one another in a relative way, which is internally consistent within the current study. Another approach is to compare SQT responses to sediment quality guideline thresholds from other studies (Burton 2002; Buchman 2008; Bay et al. 2007; Clarke and Green 1988; Borja et al. 2000 summarized in Tables 1, 3, and S4). The results of such a comparison are similar but with some differences (Table 6). Striking similarities are that both approaches identify only station R1 as degraded due to contamination. Contaminants are present at three stations (6, E, and M3) but not bioavailable. Stations FD and R2 are clearly degraded, and we have not identified why. Three stations (A, L5, and L6) with low diversity metrics are likely not degraded. The two industrial discharge sites (FD and WD) show signs of benthic degradation using both approaches, but neither approach identifies what chemicals might be causing that low sediment quality.

**Table 6:** Summary of sediment quality triad data conclusions. Symbol definitions: +  = degraded condition,− = average or above average conditions. For relative quantiles: + condition is the red symbol in in Fig. 7, and – condition is yellow and green symbols in Fig. 7. For absolute thresholds: + and – condition is based on Tables 1 and 3

Chemistry	Toxicity	Benthic	Possible conclusions	Relative quantiles	Absolute thresholds
+	+	+	Evidence of contaminant-induced degradation	R1	R1
+	−	−	Contaminants are not bioavailable	6, D, E, M3, WD	6, E, M3
−	+	+	Unmeasured contaminants or other stressor conditions are causing degradation of benthos	FD, R2	15, F, FD, R2
−	+	−	Unmeasured chemicals or conditions exist with the potential to cause degradation	B, M5, N1	A, B, N1, WD
−	−	+	Benthic response probably not due to contaminants	A, L5, L6	R3
−	−	−	No evidence of contaminant-induced degradation	8, 15, C, F, L7, M1, M2, M4, N2, R3	8, C, D, L5, L6, L7, M1, M2, M4, M5, N2

There have been previous sediment quality triad studies performed in Texas estuaries. Carr et al. (1996) found some elevated levels of chemical contaminants (PAH, total PCB, TOC, and trace elements) in Galveston Bay, indicating that localized areas were affected by anthropogenic contaminants in Galveston Bay. It was concluded that the entirety of Galveston Bay was not affected by contaminants. Localized contamination can also be concluded for this current study in the Matagorda Bay system (Fig. 6, Table 1).

Another Texas study was performed using the SQT approach, but it was limited to Lavaca Bay and focused on one chemical (mercury) (Carr et al. 2001). Mercury was known to be common near the ALCOA plant in historical studies (near stations WD and B), so it was expected that levels would be elevated (Carr et al. 2001). The current study sampled across the entirety of the Matagorda Bay system and there was a cumulative effect of multiple chemicals occurring in the estuary. Additionally, this current study indicates mercury contamination still exists in 5 stations with concentrations > TEL and 3 > ERL (Table 1, Fig. 6). Both M2 and D in Matagorda Bay had high levels, so these effects were not localized to only Lavaca Bay. Carr et al. (2001) also found that more than half of the PAHs measured exceeded ERM and PEL values for most of the stations. The current study of the entire Matagorda Bay system found no stations exceeding PEL or ERM values, and the highest PAH value was 1087 µg/kg and the other PAH chemicals measured were 50 µg/kg or less (Table 1). In the Carr et al. (2001) study in Lavaca Bay, total PAH values ranged from a low of 65.9 µg/kg to 77,000 µg/kg (Table 1). Total PCBs in the Lavaca study ranged from 0.5 µg/kg to 583 µg/kg and the current study had a high of 6.17 µg/kg (Table 1). The Carr et al. (2001) study did not address the entire bay system. In contrast, the current study has expanded the spatial assessment to encompass the entire estuarine system. Overall, there is no evidence of pollution induced degradation of the benthic community due to the targeted contaminants alone (Fig. 7).

### Benthic decline in the Matagorda Bay system

Another possible explanation for low abundance and diversity of benthic communities along the Texas Coast may be the nature of the estuaries themselves. Estuaries are naturally stressed and highly variable ecosystems with biological communities that are well adapted to variation in physio-chemical characteristics of the environment (Carr et al. 2000; Elliot and Quintino 2007; Tweedly et al. 2015). As well as being naturally stressed, estuaries can be anthropogenically stressed from a variety of sources such as industrial discharge, storm-water outfalls, non-point sources, etc. Because the Matagorda Bay system is naturally stressed (with fluctuations in nutrients, salinity, and freshwater inflow), and affected by anthropogenic stressors, it is possible that this ecosystem has reached a lower-diversity equilibrium state. For about 80 years, point source pollution (e.g., ALCOA and Formosa) has been discharged to this estuary and likely influenced benthic community structure temporally (Carr et al. 2001). Yet, this ecosystem has had decades to adapt to these anthropogenic pressures, so it could be that this is the new equilibrium (or new normal state) for this estuary.

There has been a decline in benthos abundance and biomass in the MBS from 1984 to 2019, and diversity decline in Matagorda Bay only (Montagna et al. 2025). The decline of benthos in the Matagorda Bay System is likely a result of multiple stressors. In the Matagorda Bay System, previous studies have only assessed how one of these stressors (temperature, freshwater inflow, salinity fluctuations, reduced dissolved oxygen) was causing stress to ecosystem health and benthic communities (Carr et al. 2000; Pollack et al. 2011; Montagna et al. 2025). However, to fully understand the degradation and long-term decline observed in the estuary, synergistic interactions among chemical, biological, and physical factors must be considered. Overexposure to one chemical alone does not fully explain the estuary-wide benthic decline without accounting for multiple, co-occurring environmental factors. A causal assessment framework would provide a more comprehensive approach to evaluate the relative contributions of multiple stressors and their interactions and could help identify the primary drivers of localized impacts observed in certain areas of the estuary.

### Natural stressors: salinity versus toxicity

Estuaries are subject to a large variety of natural (e.g., salinity, substrates, depth, biogeochemistry, geomorphology, and tidal characteristics) and anthropogenic stressors (e.g., pollutants, marine construction, dredging, and fishing), which combine to make it challenging to define ecological quality in these environments (Dauvin and Ruellet 2009). A unique feature of estuaries is their range of salinities, which depends on the freshwater inputs and tidal exchange with the Gulf of Mexico. Salinity levels in the Matagorda Bay system can fluctuate depending on the time of year, river discharge, and precipitation (Opdyke et al. 2025). In a 20-year study of the MBS, the salinity ranged from 2.1 to 34.2 S with a steady decrease over time (Pollack et al. 2011). In Texas, more marine influenced bays with stable salinity habitats have increased diversity, and more freshwater influenced bays with more varying salinity have decreased diversity (Van Diggelen and Montagna 2016). The current study was performed during a drought period, which could have influenced results. Perennial drought punctuated by occasional floods is common in Texas, USA, and the previous year (2021) was mostly a wet period (Opdyke et al. 2025). Precipitation, of course, influences inflow and thus salinity. The salinities were high and varied little and ranged from 26.2 to 29.9 S during sampling in May 2022. The average salinity of 28 in 2022 was 7 salinity units (i.e., 29%) higher than the long-term average of 21 for the entire system (Opdyke et al. 2025). Drought does not affect macrofauna abundance, biomass, and diversity in the MBS (Palmer and Montagna 2015). The current study provided a snapshot of environmental conditions, and these conditions are likely to vary with increases or decreases in freshwater inflow.

Riverine inputs of contaminants (e.g., PAHs, trace elements, fertilizers, animal waste, etc.) through non-point source (NPS) runoff from farm fields, streets, construction sites, etc., may play a role in estuarine degradation and benthic decline. The Matagorda Bay system has three freshwater inflow sources (Lavaca River, Tres Palacios River, and Colorado River), which can increase the amount of pollution that can enter the secondary and tertiary bays via NPS. While freshwater inflow is needed to maintain the health and sustainability of the estuarine communities, NPS can decrease benthic diversity and ecosystem health and shift trophic relationships (Boesch et al. 2001). It is tempting to attribute the high concentrations of contaminants at Station R1 to NPS. It would be informative for future sampling to be conducted in a wet season with higher freshwater inflow to compare the levels and spatial distribution of chemical contamination.

Another potential hydrological driver is the relatively long flushing time in the MBS. Over 20 years, it was estimated that the mean flushing time is 64 days ± a standard deviation of 8 days (Du et al. 2026). Flushing time is inversely proportional to freshwater inflow and thus correlated with salinity. Particles have long retention times in bays systems with long flushing times.

Overall, pollution in the Matagorda Bay System is complex because some can be categorized as localized near the industrial sites and river inlets, yet there are stations in Matagorda Bay (6, E, M3, M1, M2 and M4) exceeding TEL for As, Cd, Cu, Pb, Ni, with no obvious source of pollution. However, focusing on watershed or NPS management plans might improve or protect ecosystem health of the Lavaca-Colorado Estuary.

## Conclusions

This study found limited evidence that legacy pollutants are the dominant factor in degraded sediment quality. It is possible the long-term benthic decline was caused by chronic synergistic effects of some contaminants with low concentrations and higher contamination levels near industrial sites relative to the open bay sites. The least contaminated site was the Formosa industrial discharge site (FD), which was expected to have the most contamination. The lack of contamination may be due to solids deposited by the discharge that buried contaminants, and/or that the specific chemicals discharged were not measured in this study. The benthic community was reduced in abundance and Hill’s N1 diversity near river inlets and creeks likely due to a combination of contamination and low salinity values. Past work has shown that freshwater inflow is important to maintain coastal productivity by transporting nutrients and sediments to estuaries. But rivers and creeks also transport non-point source pollution, which could lead to chronic pollution. While industrial and wastewater discharge permitting appears to be working to limit pollutants, non-point sources should be a regulatory focus in the future. Future restoration goals should include a watershed management plan to limit non-point source pollution. The exact cause of the long-term decline in the benthic community is still uncertain but might be related to climate change and the long-term increase in temperature and decrease in dissolved oxygen (Montagna et al. 2025).

## Supplementary Information

Below is the link to the electronic supplementary material.

ESM 1 (DOCX 4.66 MB)

## Data Availability

All data can be accessed at 10.7266/9syzmzrd.
