# Supplementary material for: An assessment of contaminants and benthic condition in the Matagorda Bay system (Texas, USA)
Source: Environ Sci Pollut Res Int. 2026 Jun 15;33(19):9785–803. doi: 10.1007/s11356-026-37918-2 (PMC13294162; doi:10.1007/s11356-026-37918-2)
Supplement: Supplementary file 1 — (DOCX 4.66 MB) [file 11356_2026_37918_MOESM1_ESM.docx]

**ELECTRONIC SUPPLEMENTARY MATERIALS**

**An assessment of contaminants and benthic condition in the Matagorda Bay system (Texas, USA)**

Jasmine Caillier^a^, Paul A. Montagna*^a^, Marie E. DeLorenzo^b^, Katy W. Chung^b^, and Peter B. Key^b^

^a^ Texas A&M University-Corpus Christi, Harte Research Institute for Gulf of Mexico Studies, Corpus Christi, Texas, USA

^b^ National Oceanic and Atmospheric Administration, National Ocean Service, Charleston, South Carolina, USA

*Corresponding author: email: [Paul.Montagna@tamucc.edu](mailto:Paul.Montagna@tamucc.edu), phone: +1 361-825-2040

Submitted to: *Environmental Science and Pollution Research*

Submission Date: September 26, 2025

**SUPPLEMENTARY INTRODUCTION**

*Species Used for Toxicity Analysis*

The grass shrimp (*P. pugio*) is used as a bioindicator of anthropogenic impacts and other changes happening in the environment (Buikema et al. 1980; Key etl al 2006). Grass shrimp are one of the most sensitive organisms, but specifically they are more sensitive to heavy metals and a variety of pesticides (Anderson 1985).They are found in many estuaries along the Atlantic to Gulf coast (Heard 1982).

*Leptocheirus plumulosus,* an amphipod, is widely distributed from Cape Cod, MA to Northern Florida along the Atlantic Coast, and commonly found in marine and freshwater sediments (Manyin & Rowe 2006). Living in sediment for the entirety of their lifespan makes them more susceptible to the toxic effects of contaminated sediment ([DeWitt et al, 1992](https://www.sciencedirect.com/science/article/pii/S0141113606000286?casa_token=wO6AN6LjCBUAAAAA:C3wBCrgFTTJ9SNxLOpsm0aUt6kXPEewQD4-9iZq-BLaZuzduj626NoCOdKhDPIAcEtoN-ayYRME#bib6); [McGee et al, 1993](https://www.sciencedirect.com/science/article/pii/S0141113606000286?casa_token=wO6AN6LjCBUAAAAA:C3wBCrgFTTJ9SNxLOpsm0aUt6kXPEewQD4-9iZq-BLaZuzduj626NoCOdKhDPIAcEtoN-ayYRME#bib19)). Similarly, *N. arenaceodentata,* a polychaete, is widely distributed in shallow marine and estuarine benthic habitats of Europe, North America, and the Pacific. They are sediment dwelling organisms (in the upper 2 to 3 cm of sediment) and are known to affect the physiochemical characteristics of sediments (Reish 1972; Pesch et al, 1981).

*P. pugio* can be found in the Lavaca Colorado estuary however, *L. plumulosus* *and N. arenaceodentata* have not been found in this estuary *L. plumulosus* and *N. arenaceodentata* were selected for these tests due to the relative availability of testing protocols and the general lack of established methods for benthic toxicity testing. Although these species are not native to the estuary we sampled, the most common benthic species in the Matagorda Bay System lacks available toxicity testing protocols.

Location of companies for polychaete and amphipod sources:

- Aquatic Toxicology Support
- 1849 Charleston Beach Road W
- Bremerton, WA 98312
- Aquatic Biosystems Inc.

1300 Blue Spruce Dr. Ste. C

Fort Collins, CO, 802524

**SUPPLEMENTARY METHODS**

All raw data available at Montagna et al. 2023, <https://doi.org/10.7266/9syzmzrd>.

*Benthic Diversity Analysis*

Species diversity is calculated by replicate and by pooling all three replicate cores for each site. The four diversity metrics calculated are: richness (species number), Margalef’s richness (d), Shannon diversity, and Hill’s dominant species. Three other diversity components were calculated in addition: Pielou's Evenness index, Average Taxonomic diversity, and Average Taxonomic distinctness.

Margalef’s (1969) richness index (d) is an attempt to adjust richness (S, i.e., the number of species) for stability or persistence of a community by dividing by the logarithm of abundance (N, i.e., the total number of individuals):

**d = (S – 1)/ln N**

Hill’s diversity number 1is a measure of the effective number of species in a sample and indicates the number of abundant species (Ludwig & Reynolds 1988). It is calculated as the exponentiated form of Shannon diversity index:

**(N1 = e^H'^)**

As diversity decreases N1 will tend toward 1. The Shannon index is the average uncertainty per species in an infinite community made up of species with known proportional abundances (Shannon & Weaver 1949; Hutcheson 1970). The Shannon index is calculated as:

**(H´ = -∑[(n_i_/n) ln(n_i_/n)])**

Where *n_i_*, is the number of individuals belonging to the *i*th of S species in the sample and *n* is the total number of individuals in the sample. Hill’s N1 is used in most analyses because it is easier to interpret (Hill 1973).

The Simpson index, 1– λ is calculated as one minus the sum of the proportional abundance of each species. It is the probability that two individuals will belong to the same species and is thus a measure of dominance. The index is converted to an evenness measure by subtracting the index from one (Somerfield et al. 2008).

**1– λ = 1 – (∑ P_i_^2^)**

Pielou's evenness index (J', Pielou 1975), average taxonomic diversity (Δ), and taxonomic distinctness (Δ*) were calculated using PRIMER v7 (Clarke & Gorley 2015). Taxonomic diversity is defined as:

**Δ = [Σ Σ_i<j_ω_ij_ x_i_ x_j_]/ [N (N − 1)/2]**

Where the double summation is over all pairs of species *i* and *j,* and N = Σ_i_ x_i_, the total number of individuals in the sample Δ is the average ‘taxonomic distance apart’ of every pair of individuals in the sample, or in layman terms, the expected path length between any two individuals chosen at random (Warwick & Clarke 1995). This method is an extension of Simpson diversity by adding taxonomic relatedness.

To greater reflect the taxonomic hierarchy, dividing Δ by the Simpson index was proposed to define average taxonomic distinctness:

**Δ* = [Σ Σ_i<j_ω_ij_ x_i_ x_j_]/ [ Σ Σ_i<j_ x_i_ x_j_]**

Taxonomic distinctness is the expected taxonomic distance apart of any two individuals chosen at random from a sample, provided those two individuals are not from the same species (Warwick & Clarke 1995).

Pielou's evenness index (J') represents equitability, expressing how evenly the individuals are distributed among different species. It is defined as:

**J' = H' / H'_max_ = H' / ln S**

Where H'_max_ is the maximum possible value of Shannon diversity index (H') that can be achieved if all species were equally abundant (log S) (Warwick & Clarke 1995).

**SUPPLEMENTARY RESULTS: FIGURES AND TABLES**


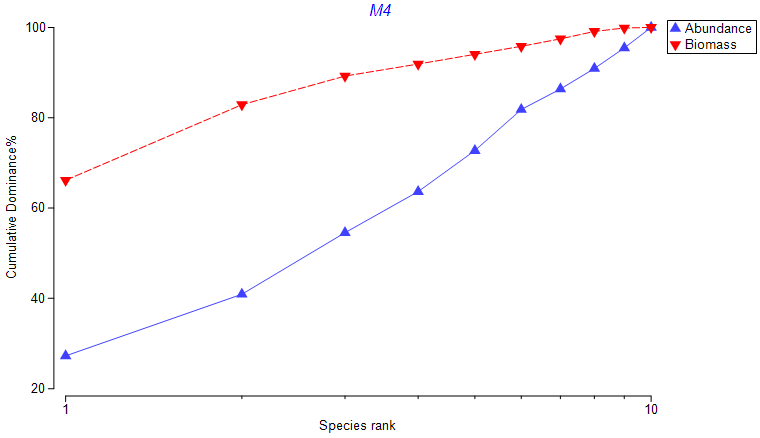

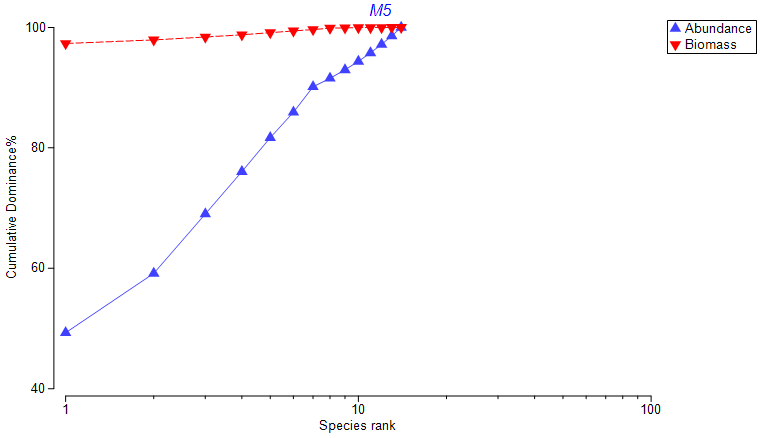

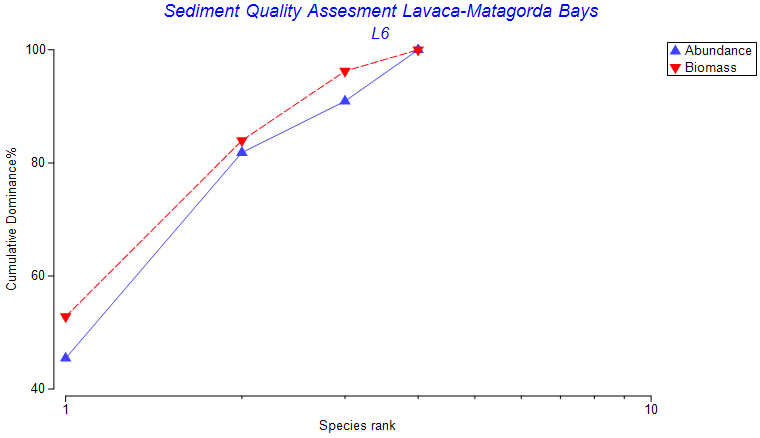


Figure S2. Abundance Biomass plot of stations describing unstressed environments, M4, M5, and L6. Abundance (blue) over Biomass (red) indicates the station is stressed and vice versa, biomass over abundance indicates unstressed environment.


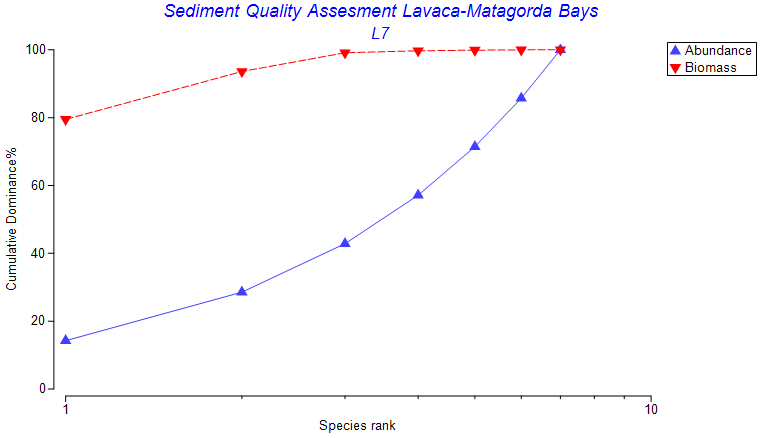

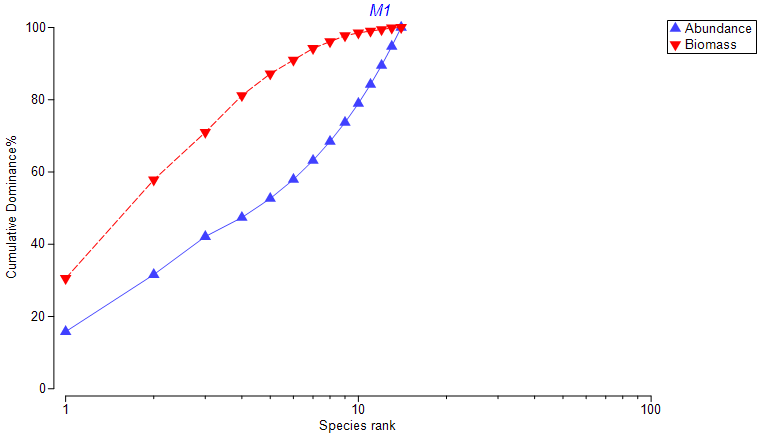

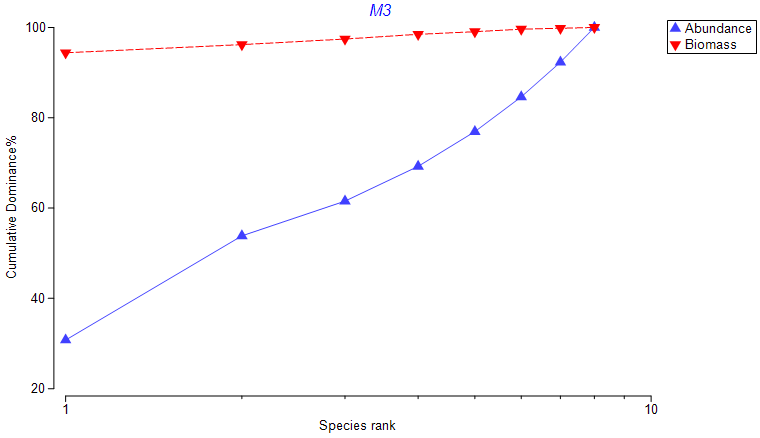

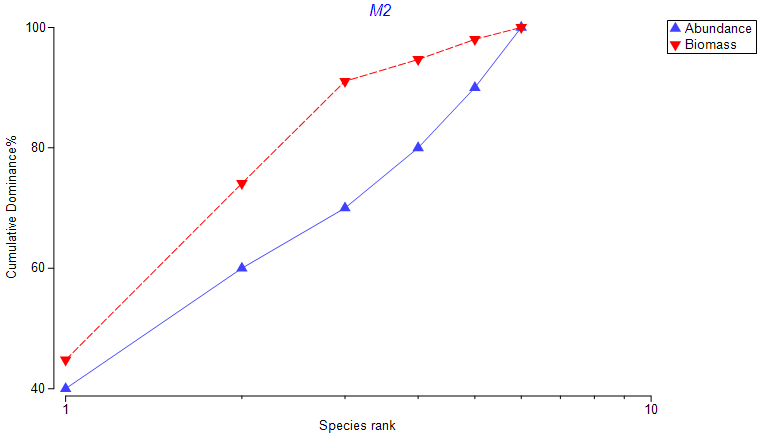


Figure S1. Abundance Biomass plot of stations describing unstressed environments, L7, M1, M2, and M3. Abundance (blue) over Biomass (red) indicates the station is stressed and vice versa, biomass over abundance indicates unstressed environment.


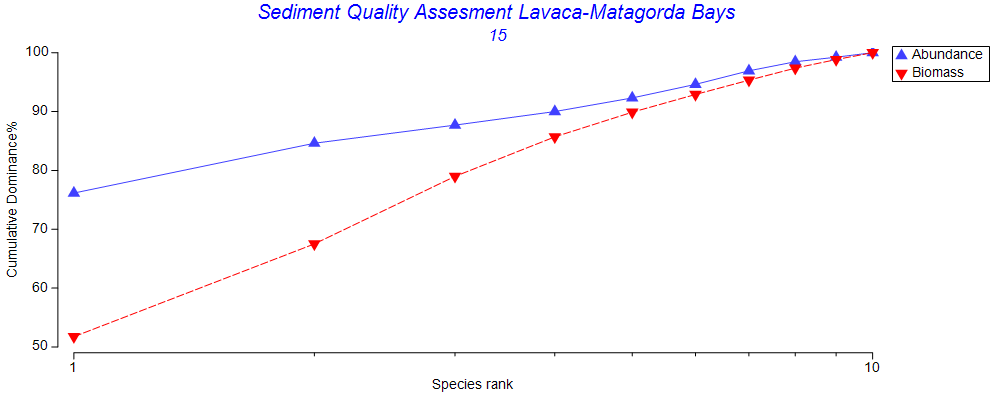

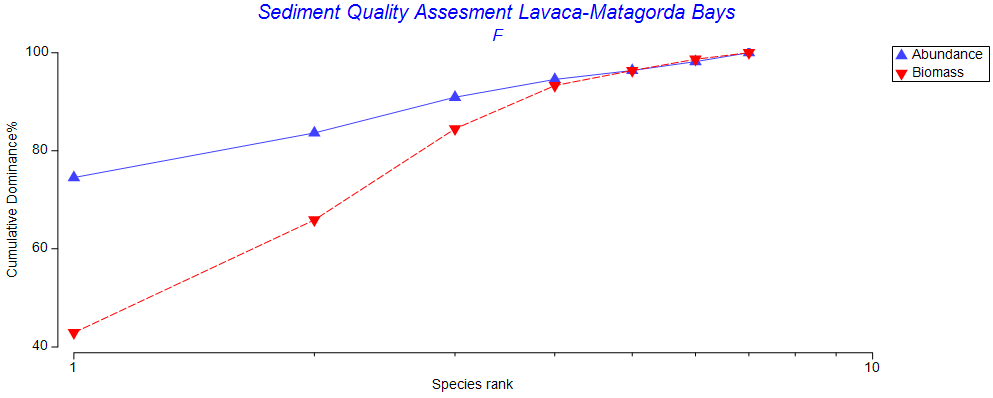

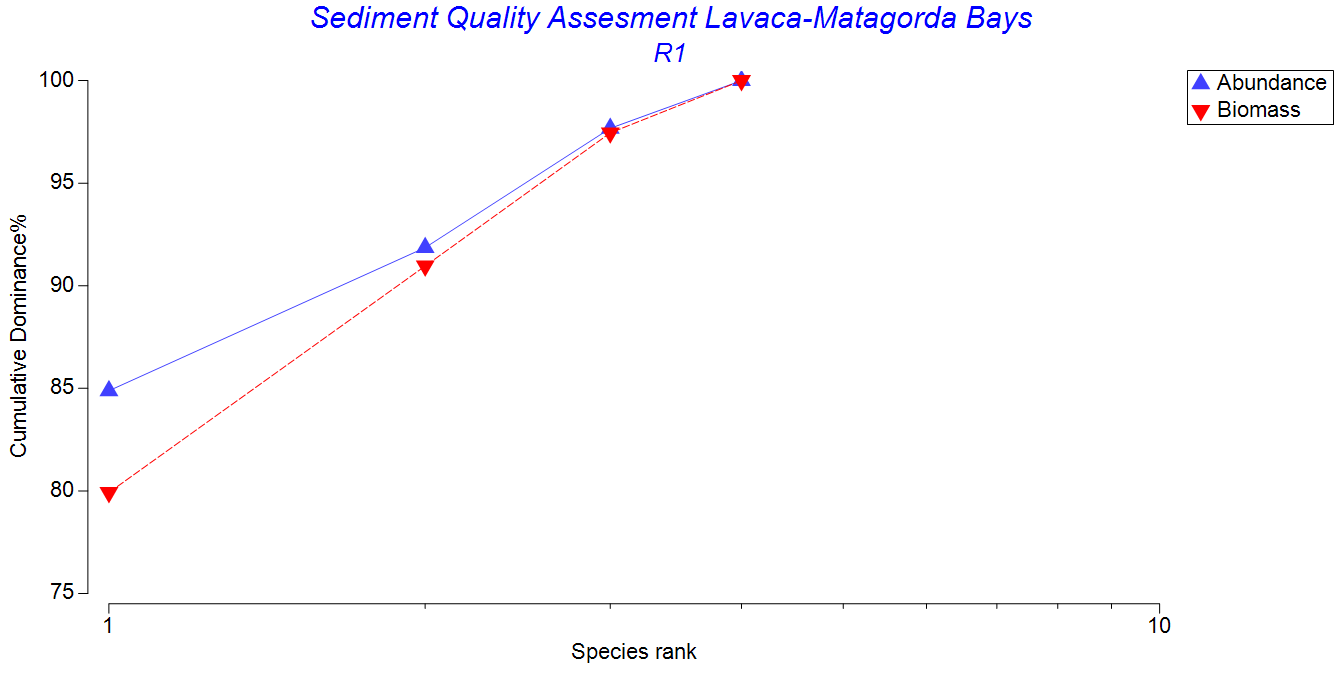

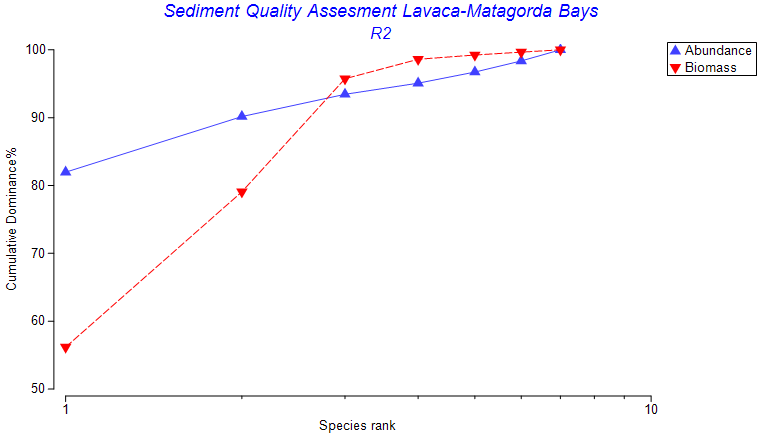


Figure S3. Abundance Biomass plot of stations (F, 15, R1, R2) describing highly stressed ecosystems indicated by current benthic conditions. Abundance (blue) over Biomass (red) indicates the station is stressed and vice versa, biomass over abundance indicates unstressed environment.


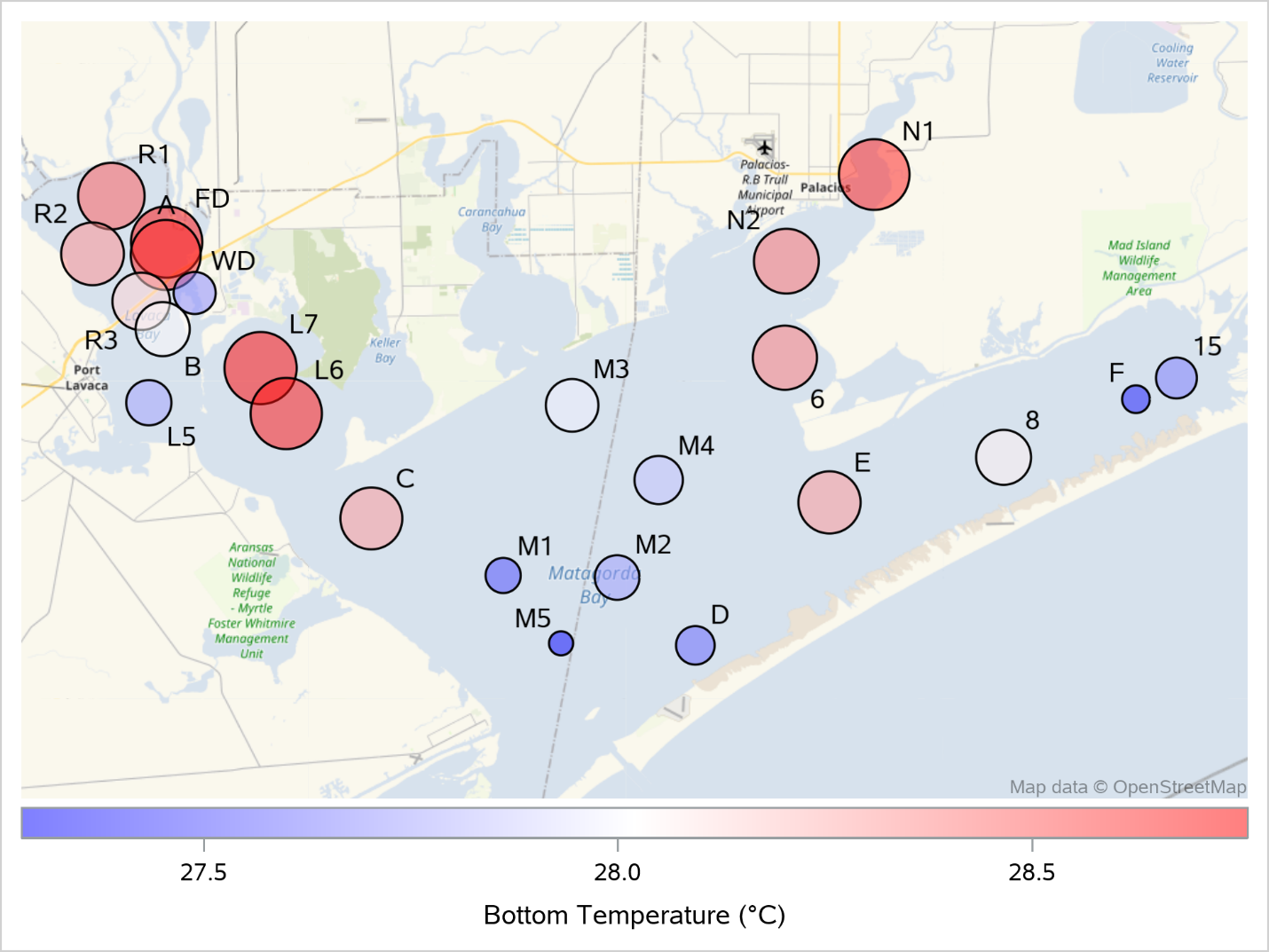


Figure S4. Map of bottom temperatures (°C) at each station.


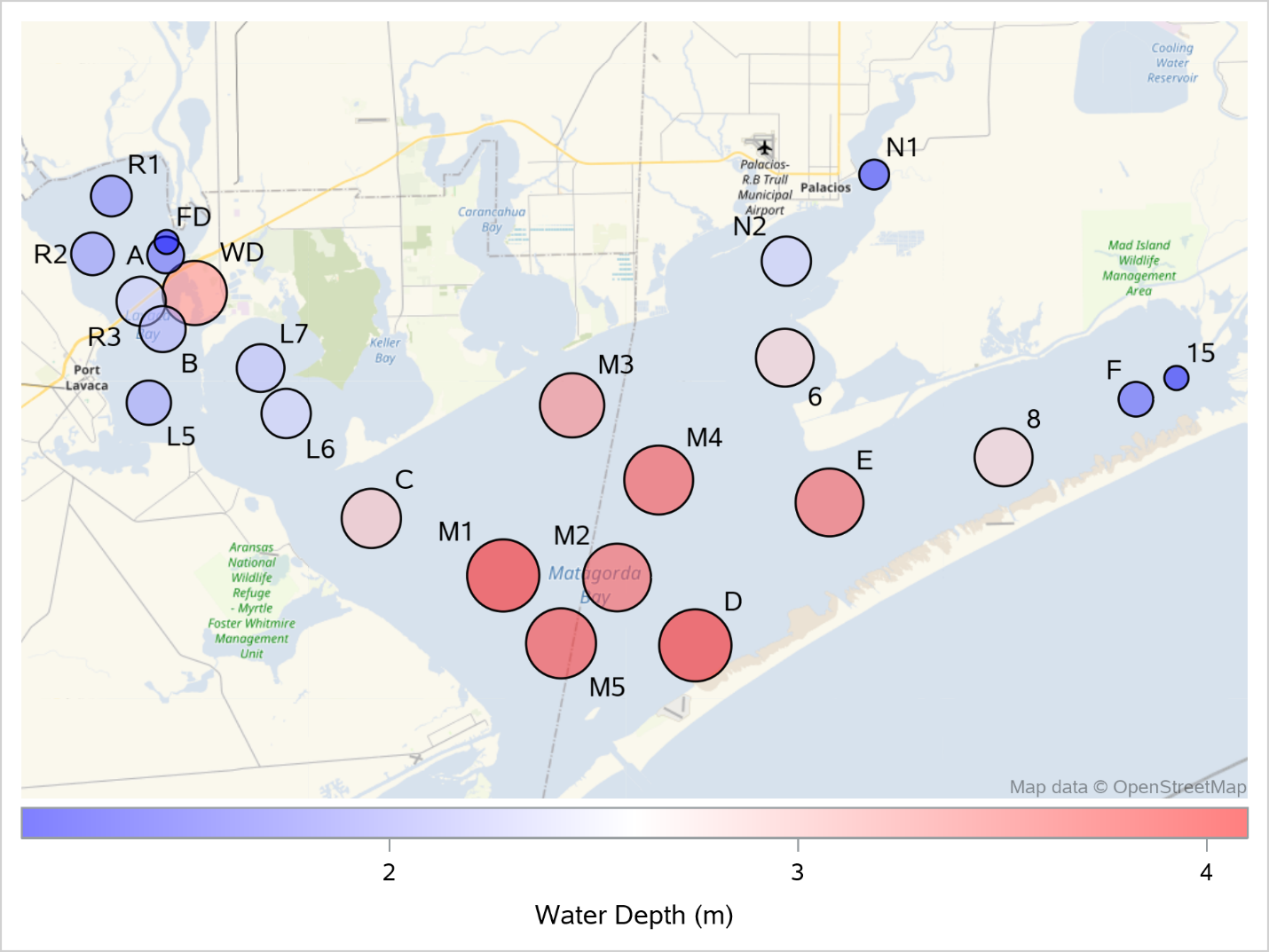


Figure S5. Map of water depths at each station.


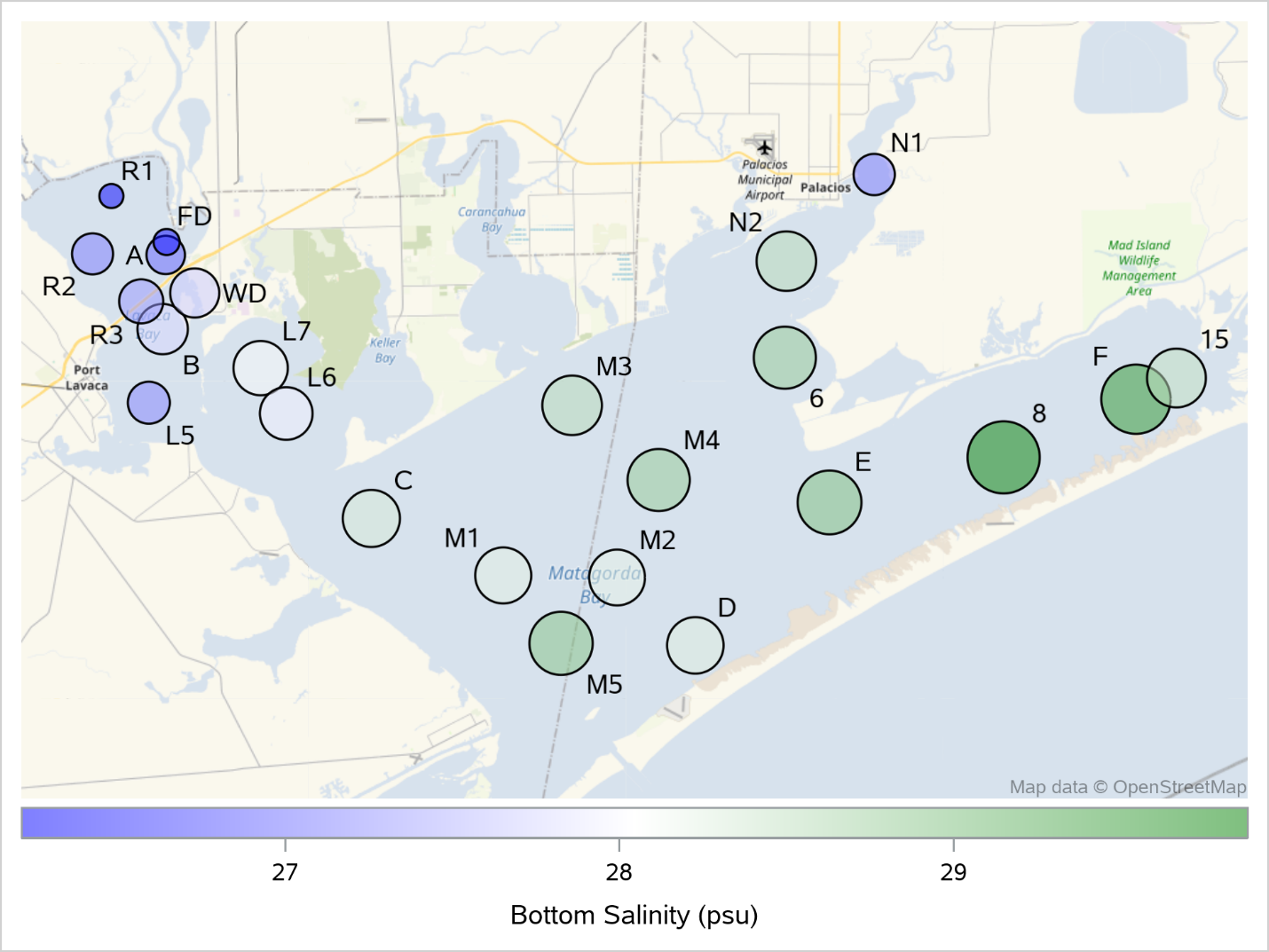


S6. Contour map of bottom salinity (S) at each station.


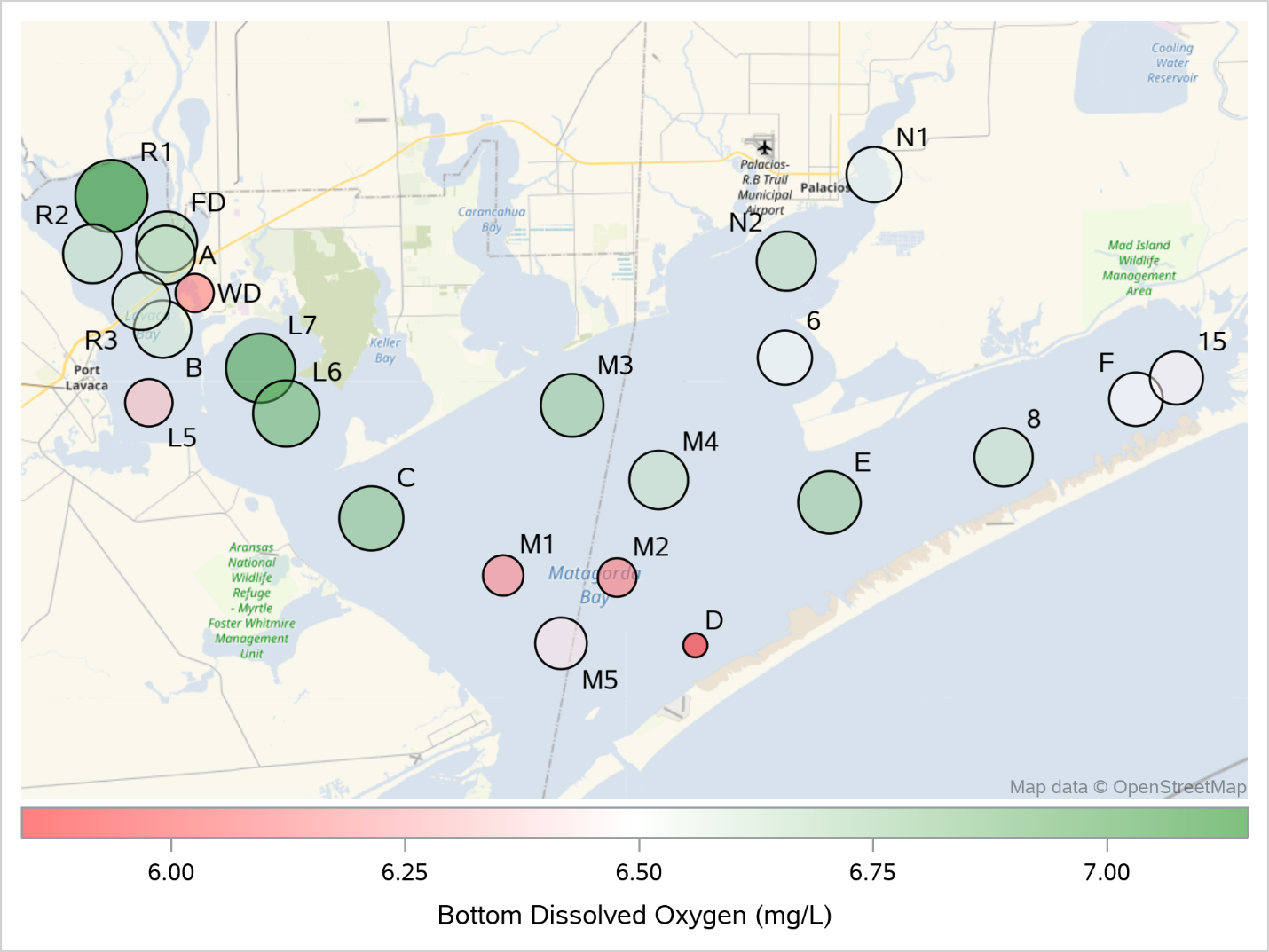


S7. Map of bottom dissolved oxygen (mg/L) at each station.


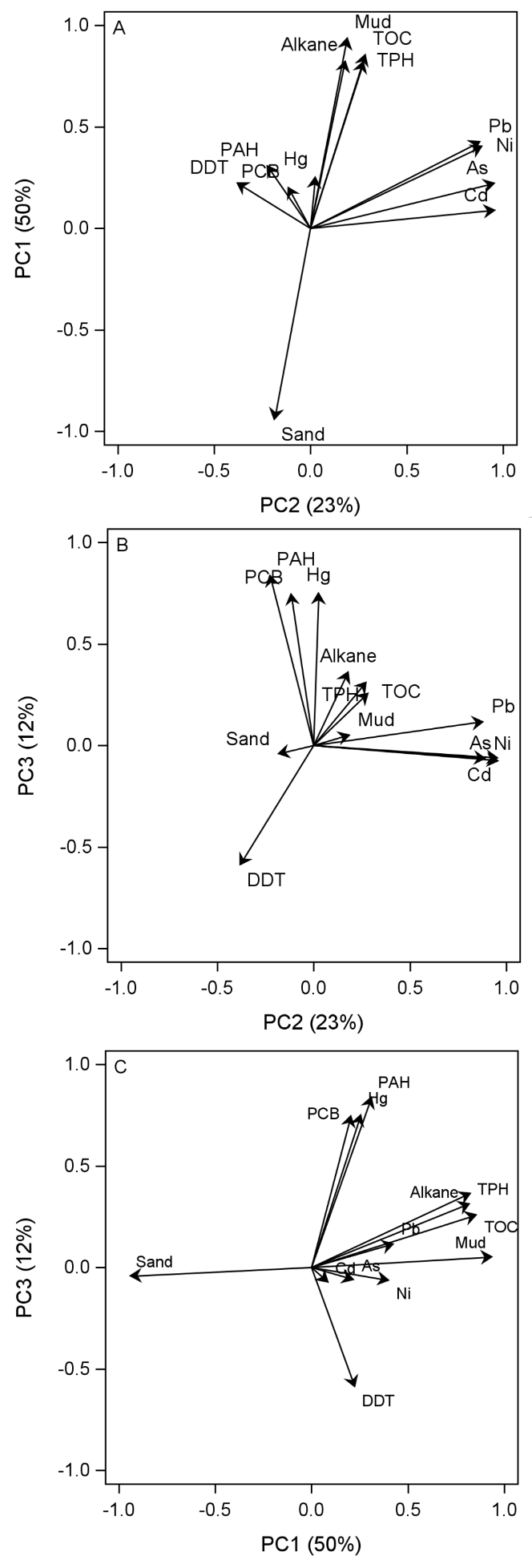
Figure S8. Rotated variable loads for PC1 versus PC2. Abbreviations: Mud = silt+clay, As = Arsenic, Cd = Cadmium, Cr = Chromium, Cu = Copper, Pb = Lead, Hg = Mercury, Ni = Nickel, Ag = Silver, Zn = Zinc, DDT = dichlorodiphenyltrichloroethanes, PAH = polycyclic aromatic hydrocarbons, PCB = polychlorinated biphenyl, and TPH = total petroleum hydrocarbons.


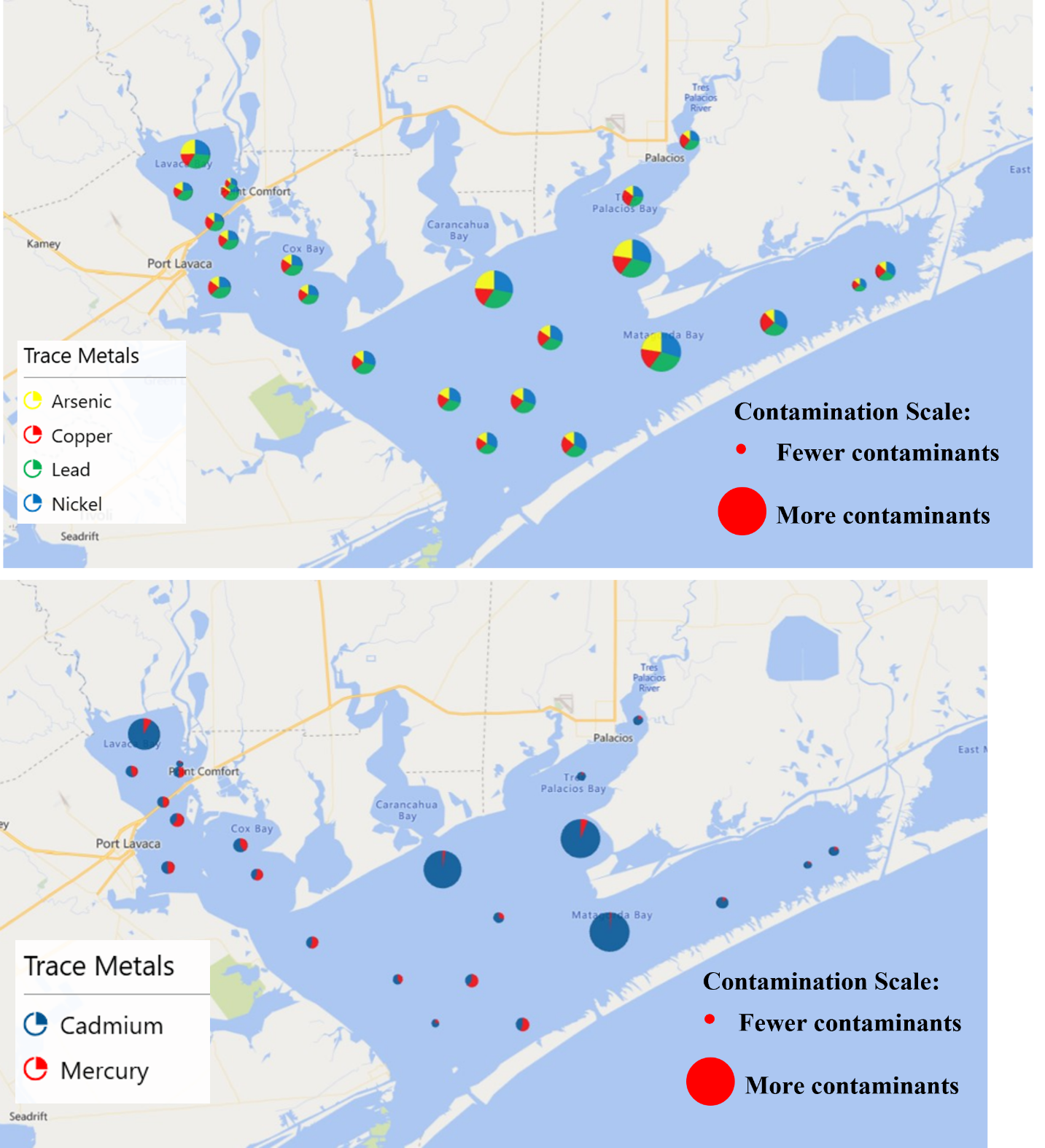


Figure S9. Maps of trace element concentrations. Top: arsenic, copper, lead, and nickel values >1 ug/kg. Bottom: cadmium and mercury values <1 ug/kg.

Table S1: Station names, locations, and comments on station choice. Abbreviations: FPC = Formosa Plastics Corporation, NFWF = National Fish and Wildlife Foundation, TCEQ = Texas Commission on Environmental Quality.

| **Station** | **Latitude** | **Longitude** | **Station Description** |
| --- | --- | --- | --- |
| 6 | 28.62479 | -96.2402 | Long term monitoring station (Montagna 2022) |
| 8 | 28.57639 | -96.1192 | Long term monitoring station |
| 15 | 28.61493 | -96.0236 | Long term monitoring station |
| A | 28.67467 | -96.58268 | Long term monitoring station |
| B | 28.63868 | -96.58437 | Long term monitoring station |
| C | 28.54672 | -96.46894 | Long term monitoring station |
| D | 28.48502 | -96.28972 | Long term monitoring station |
| E | 28.5545 | -96.2155 | Long term monitoring station |
| F | 28.60463 | -96.046 | Long term monitoring station |
| FD | 28.68096 | -96.58218 | Long term monitoring station Formosa Discharge |
| L5 | 28.60293 | -96.59201 | New Lavaca Bay station |
| L6 | 28.59769 | -96.51602 | TCEQ station – (Russell et al. 2006) |
| L7 | 28.61975 | -96.53019 | New Lavaca Bay station |
| M1 | 28.519 | -96.396 | New Matagorda Bay station |
| M2 | 28.518 | -96.333 | New Matagorda Bay station |
| M3 | 28.60166 | -96.35788 | New Matagorda Bay station |
| M4 | 28.56538 | -96.31 | New Matagorda Bay station |
| M5 | 28.486 | -96.364 | New Matagorda Bay station |
| N1 | 28.71369 | -96.19079 | NFWF station (TCEQ14680) Tres Palacios |
| N2 | 28.67166 | -96.23936 | NFWF station (TCEQ14680) Tres Palacios |
| R1 | 28.70327 | -96.61273 | FPC Monitoring station (Harris et al. 2023) |
| R2 | 28.6752 | -96.62315 | FPC Monitoring station |
| R3 | 28.65215 | -96.59625 | FPC Monitoring station |
| WD | 28.65621 | -96.56664 | Alcoa Witco Discharge monitoring station |
| Ref | 32.648394 | -80.222283 | Reference site, Leadenwah Creek, South Carolina, USA |

Table S2. MicroTox test criteria in a table format. Criteria based on Ringwood et al. (1997).

| **Criteria Number** | **EC50 Percent** | **Classification** |
| --- | --- | --- |
| 1 | 1% | Toxic |
| 2 | 0.75 % < 1% | Toxic |
| 3 | EC50's that fall below the prediction intervals established using silt normalization techniques | Toxic |
| 4 | EC50's that falls below the confidence limits established using silt normalization techniques | Toxic |
| 5 | <0.5% and Silt+Clay <20% | Toxic |
| 6 | <0.2% and Silt+Clay >20% | Toxic |

Table S3. Absolute threshold values for determining sediment quality guidelines for toxicity (i.e., survival) as a percentage of control values.

A) Threshold values for two species from Bay et al. (2007). Average survival for control is 99% for *Leptocheirus plumulosus*, and 100% for *Neanthes arenaceodentata*.

| **Species** | **Threshold (% of Control)** | | |
| --- | --- | --- | --- |
|  | **Low** | **Moderate** | **High** |
| *Leptocheirus plumulosus* | 90 | 78 | 56 |
| *Neanthes arenaceodentata* | 90 | 68 | 46 |

B) Mean survival and sediment quality guideline measures. Abbreviations: Lept = *Leptocheirus plumulosus*, Nean = *Neanthes arenaceodentata*. Measures: + = evidence of impairment for moderate and high responses only, - no evidence of impairment for low responses and above. Response color-coded as in Table A.

| **Station** | **Lept** | **Nean** | **Lept (% of Control)** | **Nean (% of Control)** | **Measure** |
| --- | --- | --- | --- | --- | --- |
| 6 | 86.00% | 92.00% | 87% | 92% | - |
| 8 | 87.00% | 84.00% | 88% | 84% | - |
| 15 | 66.00% | 92.00% | 67% | 92% | + |
| A | 76.00% | 88.00% | 77% | 88% | + |
| B | 58.00% | 80.00% | 59% | 80% | + |
| C | 85.00% | 100.00% | 86% | 100% | - |
| D | 87.00% | 84.00% | 88% | 84% | - |
| E | 85.00% | 96.00% | 86% | 96% | - |
| F | 67.00% | 96.00% | 68% | 96% | + |
| FD | 48.00% | 96.00% | 48% | 96% | + |
| L5 | 86.00% | 92.00% | 87% | 92% | - |
| L6 | 89.00% | 88.00% | 90% | 88% | - |
| L7 | 86.00% | 100.00% | 87% | 100% | - |
| M1 | 85.00% | 96.00% | 86% | 96% | - |
| M2 | 82.00% | 88.00% | 83% | 88% | - |
| M3 | 88.00% | 88.00% | 89% | 88% | - |
| M4 | 81.00% | 88.00% | 82% | 88% | - |
| M5 | 87.00% | 76.00% | 88% | 76% | - |
| N1 | 72.00% | 80.00% | 73% | 80% | + |
| N2 | 81.00% | 76.00% | 82% | 76% | - |
| R1 | 60.00% | 84.00% | 61% | 84% | + |
| R2 | 34.00% | 76.00% | 34% | 76% | + |
| R3 | 82.00% | 80.00% | 83% | 80% | - |
| WD | 75.00% | 84.00% | 76% | 84% | + |

Table S4. Absolute threshold values for determining sediment quality guidelines for benthic communities using two stress metrics AMBI and ABC.

A) Threshold for AMBI, summary of the BC and BI (Borja et al. 2000).

| **Site pollution classification** | **Biotic**  **Coefficient** | **Biotic Index** | **Dominating ecological group** | **Benthic community health** |
| --- | --- | --- | --- | --- |
| Unpolluted | 0.0 < BC ≤ 0.2 | 0 | I | Normal |
| Unpolluted | 0.2 < BC ≤ 1.2 | 1 |  | Impoverished |
| Slightly polluted | 1.2 < BC ≤ 3.3 | 2 | III | Unbalanced |
| Meanly Polluted | 3.3 < BC ≤ 4.3 | 3 |  | Transitional to polluted |
| Meanly Polluted | 4.5 < BC ≤ 5.0 | 4 | IV-V | Polluted |
| Heavily polluted | 5.0 < BC ≤ 5.5 | 5 |  | Transitional to heavy pollution |
| Heavily polluted | 5.5 < BC ≤ 6.0 | 6 | V | Heavily polluted |
| Extremely polluted | Azoic | 7 | Azoic | Azoic |

C) Summary of ABC and AMBI measures. Measures: + = evidence of impairment for moderate and high responses only, - no evidence of impairment for low responses and above. Response color-coded as in Table A. Both ABC and AMBI must meet threshold to be scored as a + measure. The ABC index threshold = positive values are unstressed communities, and <0.1 are highly stressed depending on the ABC analyses (Clarke & Green 1988; Figs S1 – S3).

| **Station** | **ABC** | **AMBI** | **Measure** |
| --- | --- | --- | --- |
| 6 | 0.45 | 2.30 | - |
| 8 | 0.15 | 3.11 | - |
| 15 | -0.32 | 4.06 | + |
| A | 0.13 | 4.00 | - |
| B | 0.33 | 3.50 | - |
| C | 0.19 | 2.94 | - |
| D | 0.28 | 3.21 | - |
| E | 0.72 | 2.25 | - |
| F | -0.19 | 4.23 | + |
| FD | 0.04 | 4.04 | + |
| L5 | 0.25 | 4.00 | - |
| L6 | 0.10 | 3.00 | - |
| L7 | 0.91 | 2.36 | - |
| M1 | 0.47 | 1.82 | - |
| M2 | 0.25 | 2.25 | - |
| M3 | 0.62 | 3.35 | - |
| M4 | 0.45 | 3.00 | - |
| M5 | 0.32 | 1.50 | - |
| N1 | 0.27 | 3.83 | - |
| N2 | 0.30 | 3.26 | - |
| R1 | -0.04 | 4.34 | + |
| R2 | -0.09 | 4.25 | + |
| R3 | 0.08 | 3.50 | + |
| WD | 0.54 | 3.90 | - |

D) Macrofauna species AMBI group classifications.

| **Phylum** | **Class** | **Family** | **Species** | **AMBI Group** |
| --- | --- | --- | --- | --- |
| Annelida | Oligochaeta | Oligochaeta | Oligochaeta | V |
| Annelida | Polychaeta | Ampharetidae | Melinna maculata | II |
| Annelida | Polychaeta | Capitellidae | Capitella capitata | V |
| Annelida | Polychaeta | Capitellidae | Mediomastus ambiseta | IV |
| Annelida | Polychaeta | Capitellidae | Notomastus latericeus | III |
| Annelida | Polychaeta | Chaetopteridae | Spiochaetopterus costarum | II |
| Annelida | Polychaeta | Cossuridae | Cossura delta | III |
| Annelida | Polychaeta | Goniadidae | Glycinde solitaria | II |
| Annelida | Polychaeta | Hesionidae | Gyptis brevipalpa | II |
| Annelida | Polychaeta | Lumbrineridae | Lumbrineris parvapedata | II |
| Annelida | Polychaeta | Magelonidae | Magelona phyllisae | II |
| Annelida | Polychaeta | Magelonidae | Magelona rosea | II |
| Annelida | Polychaeta | Nereididae | Ceratonereis irritabilis | III |
| Annelida | Polychaeta | Onuphidae | Diopatra cuprea | I |
| Annelida | Polychaeta | Orbiniidae | Haploscoloplos foliosus | II |
| Annelida | Polychaeta | Orbiniidae | Naineris laevigata | I |
| Annelida | Polychaeta | Paraonidae | Aricidea bryani | I |
| Annelida | Polychaeta | Paraonidae | Paradoneis lyra | III |
| Annelida | Polychaeta | Pilargidae | Hermundura ocularis | II |
| Annelida | Polychaeta | Pilargidae | Sigambra tentaculata | IV |
| Annelida | Polychaeta | Spionidae | Dipolydora caulleryi | III |
| Annelida | Polychaeta | Spionidae | Paraprionospio pinnata | IV |
| Annelida | Polychaeta | Spionidae | Streblospio benedicti | IV |
| Chordata | Actinopterygii | Megalopidae | Megalops atlanticus | I |
| Cnidaria | Anthozoa | Anthozoa | Anthozoa | I |
| Crustacea | Malacostraca | Ampeliscidae | Ampelisca abdita | III |
| Crustacea | Malacostraca | Apseudidae | Apseudes | III |
| Crustacea | Malacostraca | Bodotriidae | Cyclaspis varians | II |
| Crustacea | Malacostraca | Idoteidae | Edotia montosa | II |
| Crustacea | Malacostraca | Leuconidae | Leucon | II |
| Crustacea | Malacostraca | Liljeborgiidae | Listriella barnardi | II |
| Crustacea | Malacostraca | Liljeborgiidae | Listriella clymenellae | I |
| Crustacea | Malacostraca | Megalopa larvae | Megalopa larvae | I |
| Crustacea | Malacostraca | Ogyrididae | Ogyrides alphaerostris | I |
| Crustacea | Malacostraca | Pinnotheridae | Pinnixa | I |
| Echinodermata | Ophiuroidea | Amphiuridae | Microphiopholis atra | II |
| Hemichordata | Enteropneusta | Spengelidae | Schizocardium | I |
| Mollusca | Bivalvia | Hiatellidae | Hiatella arctica | II |
| Mollusca | Bivalvia | Mactridae | Mulinia lateralis | III |
| Mollusca | Bivalvia | Nuculanidae | Nuculana acuta | II |
| Mollusca | Bivalvia | Tellinidae | Macoma mitchelli | II |
| Mollusca | Gastropoda | Calyptraeidae | Crepidula | III |
| Mollusca | Gastropoda | Cylichnidae | Acteocina canaliculata | II |
| Mollusca | Gastropoda | Pyramidellidae | Eulimastoma | II |
| Nemertea | Nemertea | Nemertea | Nemertea | II |
| Phoronida | Phoronida | Phoronidae | Phoronis architecta | II |
| Platyhelminthes | Turbellaria | Turbellaria | Turbellaria | I |

Table S5: Taxonomic list of all species found with average and standard error (SE) abundance (n/m^2^) and biomass (g/m^2^).

| **Taxa Name** | | | | | **Abundance** | | **Biomass** | |
| --- | --- | --- | --- | --- | --- | --- | --- | --- |
| **Phylum** | **Class** | **Order** | **Family** | **Genus species** | **Mean** | **SE** | **Mean** | **SE** |
| Cnidaria |  |  |  |  |  |  |  |  |
|  | Anthozoa |  |  |  |  |  |  |  |
|  |  |  |  | Anthozoa (unidentified) | 7.88 | 5.45 | 0.05377 | 0.03748 |
| Platyhelminthes | |  |  |  |  |  |  |  |
|  | Turbellaria |  |  |  |  |  |  |  |
|  |  |  |  | Turbellaria (unidentified) | 3.94 | 3.94 | 0.00236 | 0.00236 |
| Nemertea |  |  |  |  |  |  |  |  |
|  |  |  |  | Nemertea (unidentified) | 94.55 | 27.88 | 0.06075 | 0.02907 |
| Phoronida |  |  |  |  |  |  |  |  |
|  |  |  | Phoronidae |  |  |  |  |  |
|  |  |  |  | Phoronis architecta | 27.58 | 14.49 | 0.00642 | 0.00388 |
| Mollusca |  |  |  |  |  |  |  |  |
|  | Gastropoda |  |  |  |  |  |  |  |
|  |  | Heterostropha |  |  |  |  |  |  |
|  |  |  | Pyramidellidae | |  |  |  |  |
|  |  |  |  | Eulimastoma sp. | 3.94 | 3.94 | 0.00004 | 0.00004 |
|  |  | Neotaeniogloassa |  |  |  |  |  |  |
|  |  |  | Calyptraeidae | |  |  |  |  |
|  |  |  |  | Crepidula sp. | 3.94 | 3.94 | 0.00154 | 0.00154 |
|  |  | Cephalaspidea |  |  |  |  |  |  |
|  |  |  | Cylichnidae |  |  |  |  |  |
|  |  |  |  | Acteocina canaliculata | 39.39 | 12.62 | 0.01655 | 0.0071 |
|  | Bivalvia |  |  |  |  |  |  |  |
|  |  | Myoida |  |  |  |  |  |  |
|  |  |  | Hiatellidae |  |  |  |  |  |
|  |  |  |  | Hiatella arctica | 11.82 | 11.82 | 0.00225 | 0.00225 |
|  |  | Nuculoida |  |  |  |  |  |  |
|  |  |  | Nuculanidae |  |  |  |  |  |
|  |  |  |  | Nuculana acuta | 3.94 | 3.94 | 0.01501 | 0.01501 |
|  |  | Veneroida |  |  |  |  |  |  |
|  |  |  | Mactridae |  |  |  |  |  |
|  |  |  |  | Mulinia lateralis | 126.06 | 34.93 | 0.08237 | 0.03774 |
|  |  |  | Tellinidae |  |  |  |  |  |
|  |  |  |  | Macoma mitchelli | 47.27 | 18.88 | 0.62767 | 0.26339 |
| Annelida |  |  |  |  |  |  |  |  |
|  | Polychaeta |  |  |  |  |  |  |  |
|  |  | Errantia |  |  |  |  |  |  |
|  |  |  | Pilargidae |  |  |  |  |  |
|  |  |  |  | Hermundura ocularis | 11.82 | 11.82 | 0.00638 | 0.00638 |
|  |  |  |  | Sigambra tentaculata | 11.82 | 11.82 | 0.0076 | 0.0076 |
|  |  |  | Hesionidae |  |  |  |  |  |
|  |  |  |  | Gyptis brevipalpa | 11.82 | 6.52 | 0.00355 | 0.00241 |
|  |  |  | Nereididae |  |  |  |  |  |
|  |  |  |  | Ceratonereis irritabilis | 3.94 | 3.94 | 0.00059 | 0.00059 |
|  |  |  | Goniadidae |  |  |  |  |  |
|  |  |  |  | Glycinde solitaria | 122.12 | 24.46 | 0.02612 | 0.00877 |
|  |  |  | Onuphidae |  |  |  |  |  |
|  |  |  |  | Diopatra cuprea | 11.82 | 8.65 | 0.01355 | 0.0094 |
|  |  |  | Lumbrineridae | |  |  |  |  |
|  |  |  |  | Lumbrineris parvapedata | 7.88 | 5.45 | 0.00087 | 0.0006 |
|  |  | Canalipalpata |  |  |  |  |  |  |
|  |  |  | Spionidae |  |  |  |  |  |
|  |  |  |  | Dipolydora caulleryi | 31.52 | 27.69 | 0.00362 | 0.00354 |
|  |  |  |  | Paraprionospio pinnata | 145.76 | 42.58 | 0.18015 | 0.05639 |
|  |  |  |  | Streblospio benedicti | 102.43 | 36.85 | 0.00414 | 0.00163 |
|  |  |  | Magelonidae |  |  |  |  |  |
|  |  |  |  | Magelona phyllisae | 7.88 | 7.88 | 0.00559 | 0.00559 |
|  |  |  |  | Magelona rosea | 3.94 | 3.94 | 0.00122 | 0.00122 |
|  |  |  | Chaetopteridae | |  |  |  |  |
|  |  |  |  | Spiochaetopterus costarum | 15.76 | 7.35 | 0.01359 | 0.00789 |
|  |  |  | Ampharetidae | |  |  |  |  |
|  |  |  |  | Melinna maculata | 3.94 | 3.94 | 0.01619 | 0.01619 |
|  |  | Sedentaria |  |  |  |  |  |  |
|  |  |  | Capitellidae |  |  |  |  |  |
|  |  |  |  | Capitella capitata | 3.94 | 3.94 | 0.00162 | 0.00162 |
|  |  |  |  | Mediomastus ambiseta | 1847.6 | 499.52 | 0.1551 | 0.04623 |
|  |  |  |  | Notomastus latericeus | 3.94 | 3.94 | 0.00134 | 0.00134 |
|  |  |  | Cossuridae |  |  |  |  |  |
|  |  |  |  | Cossura delta | 157.58 | 39.29 | 0.02084 | 0.00615 |
|  |  |  | Orbiniidae |  |  |  |  |  |
|  |  |  |  | Haploscoloplos foliosus | 55.15 | 25.4 | 0.08623 | 0.04987 |
|  |  |  |  | Naineris laevigata | 3.94 | 3.94 | 0.00232 | 0.00232 |
|  |  |  | Paraonidae |  |  |  |  |  |
|  |  |  |  | Aricidea bryani | 15.76 | 9.29 | 0.02056 | 0.012 |
|  |  |  |  | Paradoneis lyra | 3.94 | 3.94 | 0.00008 | 0.00008 |
|  | Oligochaeta |  |  |  |  |  |  |  |
|  |  |  |  | Oligochaeta (unidentified) | 23.64 | 14.23 | 0.00016 | 0.00009 |
| Crustacea |  |  |  |  |  |  |  |  |
|  | Malacostraca | |  |  |  |  |  |  |
|  |  | Decapoda (Natantia) | |  |  |  |  |  |
|  |  |  | Ogyrididae |  |  |  |  |  |
|  |  |  |  | Ogyrides alphaerostris | 15.76 | 7.35 | 0.01627 | 0.0101 |
|  |  | Decapoda (Reptantia) | |  |  |  |  |  |
|  |  |  | Pinnotheridae | |  |  |  |  |
|  |  |  |  | Pinnixa sp. | 3.94 | 3.94 | 0.01627 | 0.01627 |
|  |  |  |  | Megalopa larvae | 3.94 | 3.94 | 0.0011 | 0.0011 |
|  |  | Cumacea |  |  |  |  |  |  |
|  |  |  | Bodotriidae |  |  |  |  |  |
|  |  |  |  | Cyclaspis varians | 3.94 | 3.94 | 0.00024 | 0.00024 |
|  |  |  | Leuconidae |  |  |  |  |  |
|  |  |  |  | Leucon sp. | 23.64 | 10.26 | 0.00102 | 0.00059 |
|  |  | Amphipoda |  |  |  |  |  |  |
|  |  |  | Ampeliscidae |  |  |  |  |  |
|  |  |  |  | Ampelisca abdita | 86.67 | 48.6 | 0.00382 | 0.00201 |
|  |  |  | Liljeborgiidae |  |  |  |  |  |
|  |  |  |  | Listriella barnardi | 23.64 | 13.04 | 0.00205 | 0.00111 |
|  |  |  |  | Listriella clymenellae | 3.94 | 3.94 | 0.00024 | 0.00024 |
|  |  | Isopoda |  |  |  |  |  |  |
|  |  |  | Idoteidae |  |  |  |  |  |
|  |  |  |  | Edotia montosa | 3.94 | 3.94 | 0.00047 | 0.00047 |
|  |  | Tanaidacea |  |  |  |  |  |  |
|  |  |  | Apseudidae |  |  |  |  |  |
|  |  |  |  | Apseudes sp. A | 3.94 | 3.94 | 0.00374 | 0.00374 |
| Echinodermata | |  |  |  |  |  |  |  |
|  | Ophiuroidea |  |  |  |  |  |  |  |
|  |  | Ophiurida |  |  |  |  |  |  |
|  |  |  | Amphiuridae |  |  |  |  |  |
|  |  |  |  | Microphiopholis atra | 7.88 | 5.45 | 0.02045 | 0.01702 |
| Chordata |  |  |  |  |  |  |  |  |
|  | Actinopterygii | |  |  |  |  |  |  |
|  |  | Elopiformes |  |  |  |  |  |  |
|  |  |  | Megalopidae |  |  |  |  |  |
|  |  |  |  | Megalops atlanticus | 3.94 | 3.94 | 0.00036 | 0.00036 |
| Hemichordata | |  |  |  |  |  |  |  |
|  | Enteropneusta | |  |  |  |  |  |  |
|  |  | Order unassigned |  |  |  |  |  |  |
|  |  |  | Spengelidae |  |  |  |  |  |
|  |  |  |  | Schizocardium sp. | 177.28 | 141.74 | 1.2306 | 1.00016 |
| **Total** |  |  |  |  | **3340.65** | **1202.19** | **2.74** | **1.7** |

Table S6. Environmental variables that explain macrofauna community structure patterns using the biota and/or environment matching (BIOENV) procedure in Primer-e. Variables included in the analysis were all macrofauna species against As, Cd, Hg, Pb, Ni, Sand, Mud, TOC, TPH, Alkane, PAH, DDT, and PCB.

*Best results for each number of variables:*

| **No.Vars** | **Corr.** | **Selections** |
| --- | --- | --- |
| 1 | 0.141 | Ni |
| 2 | 0.163 | Cd, Ni |
| 3 | 0.161 | Hg, Ni, TPH |
| 4 | 0.159 | Hg, Ni, Sand, TPH |
| 5 | 0.154 | Hg, Ni, Sand, TPH, DDT |

*Best results overall:*

| **No.Vars** | **Corr.** | **Selections** |
| --- | --- | --- |
| 2 | 0.163 | Cd, Ni |
| 3 | 0.161 | Hg, Ni, TPH |
| 4 | 0.159 | Hg, Ni, Sand, TPH |
| 4 | 0.154 | Ni, Sand, TPH, DDT |
| 5 | 0.154 | Hg, Ni, Sand, TPH, DDT |
| 3 | 0.153 | As, Cd, Ni |
| 5 | 0.152 | Ni, Sand, TOC, TPH, DDT |
| 4 | 0.151 | Hg, Ni, TOC, TPH |
| 4 | 0.150 | Ni, Sand, TOC, DDT |
| 5 | 0.148 | Hg, Ni, Sand, TOC, TPH |

Table S7. Spearman correlation (r) and probability level (p). Bolded p values are <0.05. A) benthic community metrics. Abbreviations: S = Richness, N = number of organisms n/m^2^, d = Margalef richness, J' = Pielou’s Evenness, H' = Shannon’s diversity, N1 = Hill’s diversity number 1, ABC = ABC index (Table S2), Survival = Average survival among all species (toxicity). B) Benthic metrics correlated to environmental principal components (PC) (Fig. S4). PC1 interpreted as sediment texture (sand) vs. hydrocarbon concentrations (TPH and alkanes), PC2 interpreted as metal concentrations, and PC3 interpreted as PAH, PCB, and Hg vs. DDT concentrations.

| **A)** | **S** | | **N** | | **d** | | **J'** | | **H'** | | **N1** | | **ABC** | |
| --- | --- | --- | --- | --- | --- | --- | --- | --- | --- | --- | --- | --- | --- | --- |
|  | **r** | **p** | **r** | **p** | **r** | **p** | **r** | **p** | **r** | **p** | **r** | **p** | **r** | **p** |
| **S** | 1 |  |  |  |  |  |  |  |  |  |  |  |  |  |
| **N** | 0.438 | **0.0321** | 1 |  |  |  |  |  |  |  |  |  |  |  |
| **d** | 0.970 | **<.0001** | 0.240 | 0.2585 | 1 |  |  |  |  |  |  |  |  |  |
| **J** | 0.149 | 0.4859 | -0.702 | **0.0001** | 0.355 | 0.0889 | 1 |  |  |  |  |  |  |  |
| **H** | 0.726 | **<.0001** | -0.184 | 0.3895 | 0.851 | **<.0001** | 0.743 | **<.0001** | 1 |  |  |  |  |  |
| **N1** | 0.726 | **<.0001** | -0.184 | 0.3895 | 0.851 | **<.0001** | 0.743 | **<.0001** | 1 | **<.0001** | 1 |  |  |  |
| **ABC** | 0.148 | 0.4891 | -0.611 | **0.0015** | 0.333 | 0.1122 | 0.879 | **<.0001** | 0.650 | **0.0006** | 0.650 | **0.0006** |  |  |
| **Survival** | 0.201 | 0.3465 | -0.531 | **0.0076** | 0.330 | 0.1157 | 0.603 | **0.0018** | 0.530 | **0.0077** | 0.530 | **0.0077** | 0.502 | **0.0125** |

| **B)** | **S** | | **N** | | **d** | | **J'** | | **H'** | | **N1** | | **ABC** | |
| --- | --- | --- | --- | --- | --- | --- | --- | --- | --- | --- | --- | --- | --- | --- |
|  | **r** | **p** | **r** | **p** | **r** | **p** | **r** | **p** | **r** | **p** | **r** | **p** | **r** | **p** |
| **PC1** | 0.164 | 0.4430 | -0.355 | 0.0884 | 0.286 | 0.1753 | 0.637 | **0.0008** | 0.485 | **0.0162** | 0.537 | **0.0069** | 0.485 | **0.0162** |
| **PC2** | 0.067 | 0.7541 | -0.334 | 0.1112 | 0.192 | 0.3683 | 0.586 | **0.0026** | 0.425 | **0.0383** | 0.490 | **0.0150** | 0.425 | **0.0383** |
| **PC3** | -0.140 | 0.5148 | -0.524 | **0.0086** | -0.006 | 0.9775 | 0.460 | **0.0237** | 0.250 | 0.2379 | 0.270 | 0.2012 | 0.250 | 0.2379 |

**SUPPLEMENTARY REFERENCES**

Anderson, G.S., 1985. *Species profiles: Life histories and environmental requirements of coastal fishes and invertebrates (Gulf of Mexico): Grass shrimp* (Vol. 82). The Service.

Bay, S, D. Greenstein, and D. Young. 2007. Evaluation of methods for measuring sediment toxicity in California bays and estuaries. Technical Report 503, Southern California Coastal Water Research Project, Costa Mesa, CA, USA, 41 p, and 2 appendices.

Borja, A, J. Franco, and V. Perez. 2000. Marine Pollution Bulletin 40(12): 1100-1114.

Buikema A, Jr, Niederlehner B, Cairns J. 1980. Use of grass shrimp in toxicity tests. In: Buikema JA, Cairns JJ, editors. Aquatic Invertebrates Bioassays ASTM STP 715. Philadelphia, PA: American Society for Testing and Materials. p 155–173

Clarke, K.R., and R.N. Gorley. 2015. *PRIMER v7: User Manual/Tutorial*. PRIMER-E: Plymouth.

Clarke, K.R., and R.H. Green. 1988. Statistical design and analysis for a ‘biological effects’ study. Mar Ecol Prog Ser 46: 213-226. https://www.jstor.org/stable/24827586

DeWitt, T. H., Redmond, M. S., Sewall, J. E., & Swartz, R. C. 1992. *Development of a chronic sediment toxicity test for marine benthic amphipods* (No. PB-93-196020/XAB; CBP/TRS-89/93). Environmental Protection Agency, Newport, OR (United States). Environmental Research Lab.

Heard, R.W. 1982. Guide to common tidal marsh invertebrates of the Northeastern Gulf of Mexico. Mississippi-Alabama Sea Grant Consortium. MASGP79-004.

Hill, M.O. 1973. Diversity and evenness: a unifying notation and its consequences. *Ecology* 54: 427-432. <https://doi.org/10.2307/1934352>

Hutcheson K. 1970. A test for comparing diversities based on the Shannon formula. *Journal of Theoretical Biology* 29: 151–154.

Key, P.B., Wirth, E.F. and Fulton, M.H., 2006. A review of grass shrimp, Palaemonetes spp., as a bioindicator of anthropogenic impacts. *Environmental Bioindicators*, *1*(2), pp.115-128.

Ludwig J.A. and J.F. Reynolds. 1988. *Statistical Ecology*. John Wiley and Sons, New York.

McGee, B.L., Schlekat, C.E., & Reinharz, E. 1993. Assessing sublethal levels of sediment contamination using the estuarine amphipod *Leptocheirus plumulosus*. *Environmental Toxicology and Chemistry: An International Journal*, *12*(3), 577-587.

Margalef, S.R. 1969. Diversity and stability: a practical proposal and a model of interdependence. *Clearinghouse for Federal Scientific and Technical Information* 22: 25-37. <https://digital.csic.es/bitstream/10261/166352/3/Margalef_1969_preprint.pdf>

Montagna, P.A., J. Caillier, M. E. DeLorenzo, and P. Key. 2023. Sediment Quality Triad (SQT) Assessment Survey of Lavaca and Matagorda Bays. Distributed by: Gulf of Mexico Research Initiative Information and Data Cooperative (GRIIDC), Harte Research Institute, Texas A&M University–Corpus Christi. <https://doi.org/10.7266/9syzmzrd>

Pielou, E.C. 1975. *Ecological Diversity*. Wiley, New York.

Pesch, G. G., Pesch, C. E., & Malcolm, A. R. 1981. *Neanthes arenaceodentata*, a cytogenetic model for marine genetic toxicology. *Aquatic toxicology*, *1*(5-6), 301-311.

Reish, D.J. 1972. The use of marine invertebrates as indicators of varying degrees of marine pollution. *Marine pollution and sea life*, 203-207.

Russell, M.J., P.A. Montagna, and R.D. Kalke. 2006. The effect of freshwater inflow on net ecosystem metabolism in Lavaca Bay, Texas. *Estuarine, Coastal and Shelf Science* 68: 231-244. <http://doi.org/10.1016/j.ecss.2006.02.005>

Shannon, C.E. and W. Weaver. 1949. *The Mathematical Theory of Communication*. University of Illinois Press. Urbana, IL.

Somerfield, P.J, K.R. Clarke, and R.M. Warwick. 2008 Simpson index. *Encyclopedia of Ecology* 3252-3255. <https://doi.org/10.1016/B978-008045405-4.00133-6>

Warwick, R.M., and K.R. Clarke. 1995. New 'biodiversity' measures reveal a decrease in taxonomic distinctness with increasing stress. *Marine Ecology Progress Series* 129: 301-305. <http://doi.org/10.3354/meps129301>
